# Supplementary material for: Transcriptome 3′end organization by PCF11 links alternative polyadenylation to formation and neuronal differentiation of neuroblastoma
Source: Nat Commun. 2018 Dec 14;9:5331. doi: 10.1038/s41467-018-07580-5 (PMC6294251; doi:10.1038/s41467-018-07580-5)
Supplement: Supplementary file 2 — Supplementary Information [file 41467_2018_7580_MOESM2_ESM.pdf]

Transcriptome 3'end organization by PCF11 links alternative polyadenylation to formation and neuronal differentiation of neuroblastoma

Ogorodnikov et al.

Supplementary Figure 1

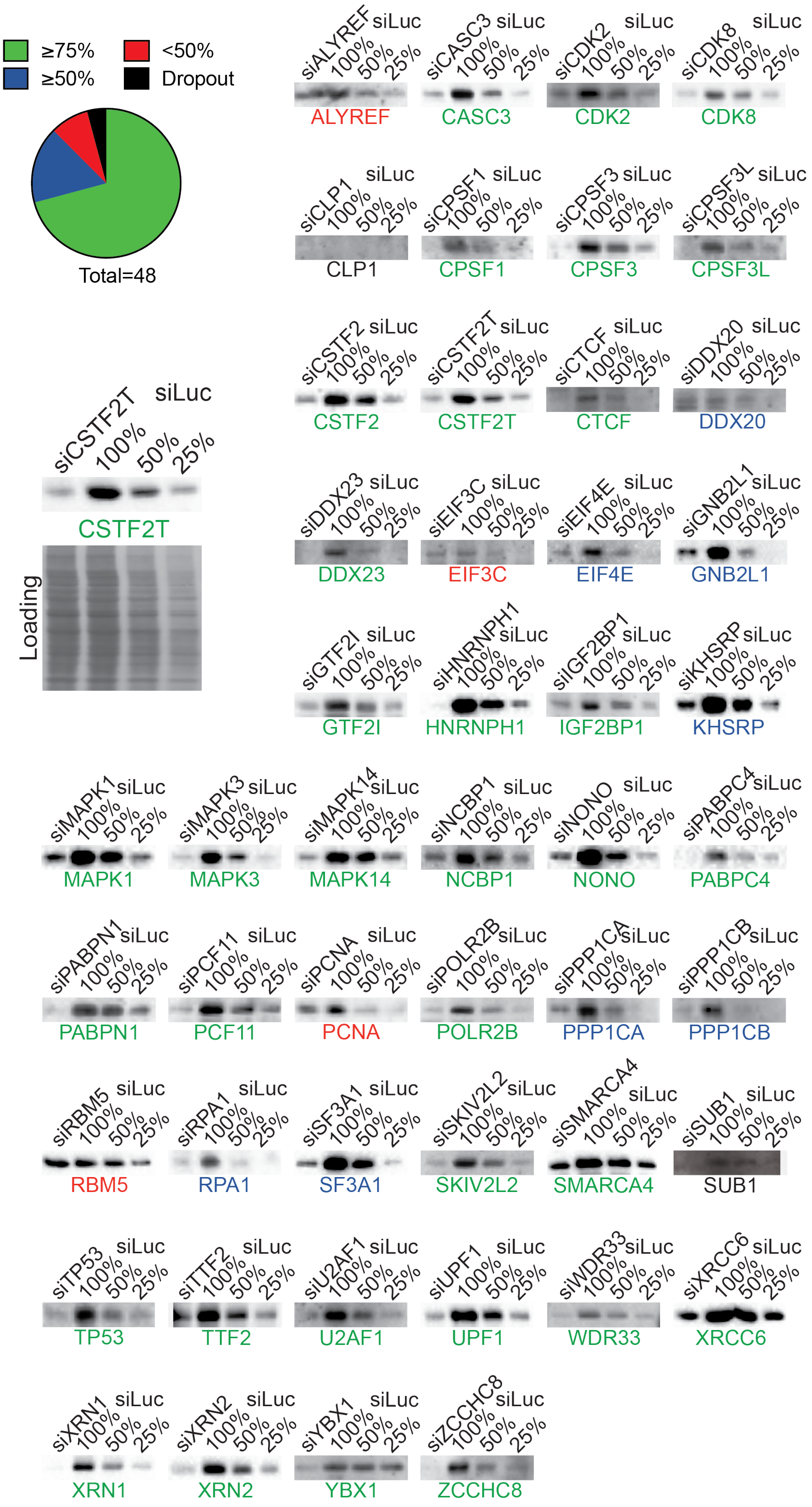

Targeting drivers of transcriptome 3'end diversity (TREND) in a model of neuroblastoma.

Depletion of 174 putative TREND-regulators with a custom siRNA library in BE(2)-C cells. Knockdown efficiency for 48 randomly selected candidates (western blotting) confirming a successful depletion (down to at least 25%) for more than 70% of the putative TREND-regulators (shown loading control applies to all knockdowns, whole-protein stain).

Supplementary Figure 2

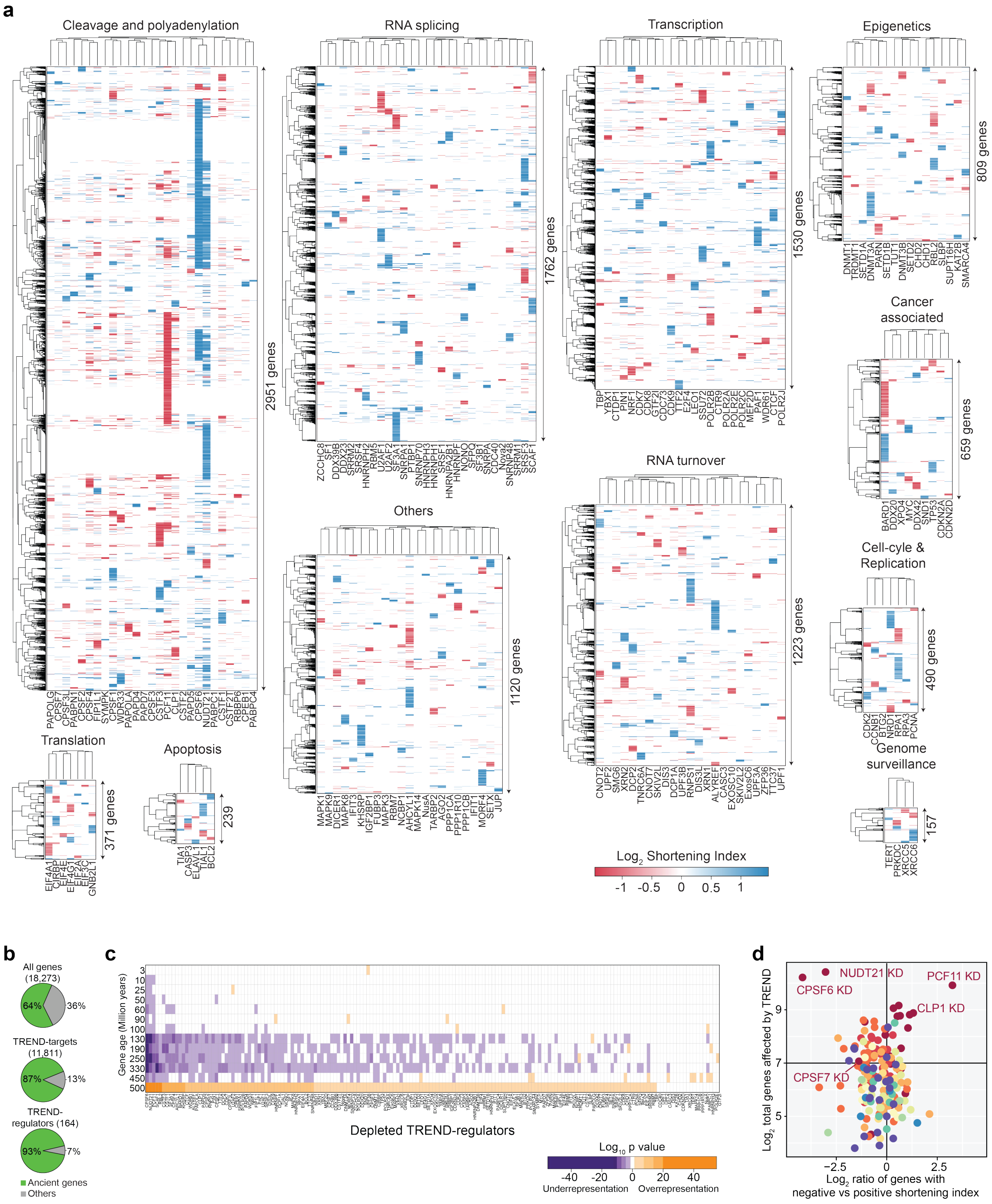

TREND is phylogenetically conserved and controlled by various mechanisms affecting RNA life.

a Heat maps of clustered TREND-affected genes (number on the right, y-axis, scaling of the graphs corresponds to number of TREND-affected genes) grouped per functional category of depleted TREND-regulators (x-axes; hierarchical clustering according to shortening index is based on Pearson's correlation coefficient and complete linkage method). Identity of genes and TREND-signatures are displayed in detail in the TREND-DB web explorer ([http://shiny.imbei.uni-mainz.de:3838/trend-db], see also **Supplementary Table 2**). **b** Age indexing of genes with discovered TREND-isoforms (middle panel) and screened TREND-

regulators (lower panel) implying a high conservation of TREND (human gene age assignments obtained from[1]; 'ancient genes' are genes with gene age >450 million years). **c** Enrichment analysis of age index of TREND-affected genes upon siRNA-depletion of 174 TREND-regulators revealing that dynamic changes at the RNA 3'end are mostly found among ancient genes (orange and purple depict significant over- and underrepresentation, respectively, of genes of a particular age group (y-axis); depleted TREND-regulators are shown on the x-axis; hyper-geometric test enrichment p-values; only p-values below 0.05 are coloured). **d** Components of the CFIm (NUDT21 and CPSF6) and CFIIm complexes (PCF11) pervasively regulate TREND in neuroblastoma in a unidirectional manner (total number of genes affected by TREND (y-axis), **Supplementary Table 2**).

Supplementary Figure 3

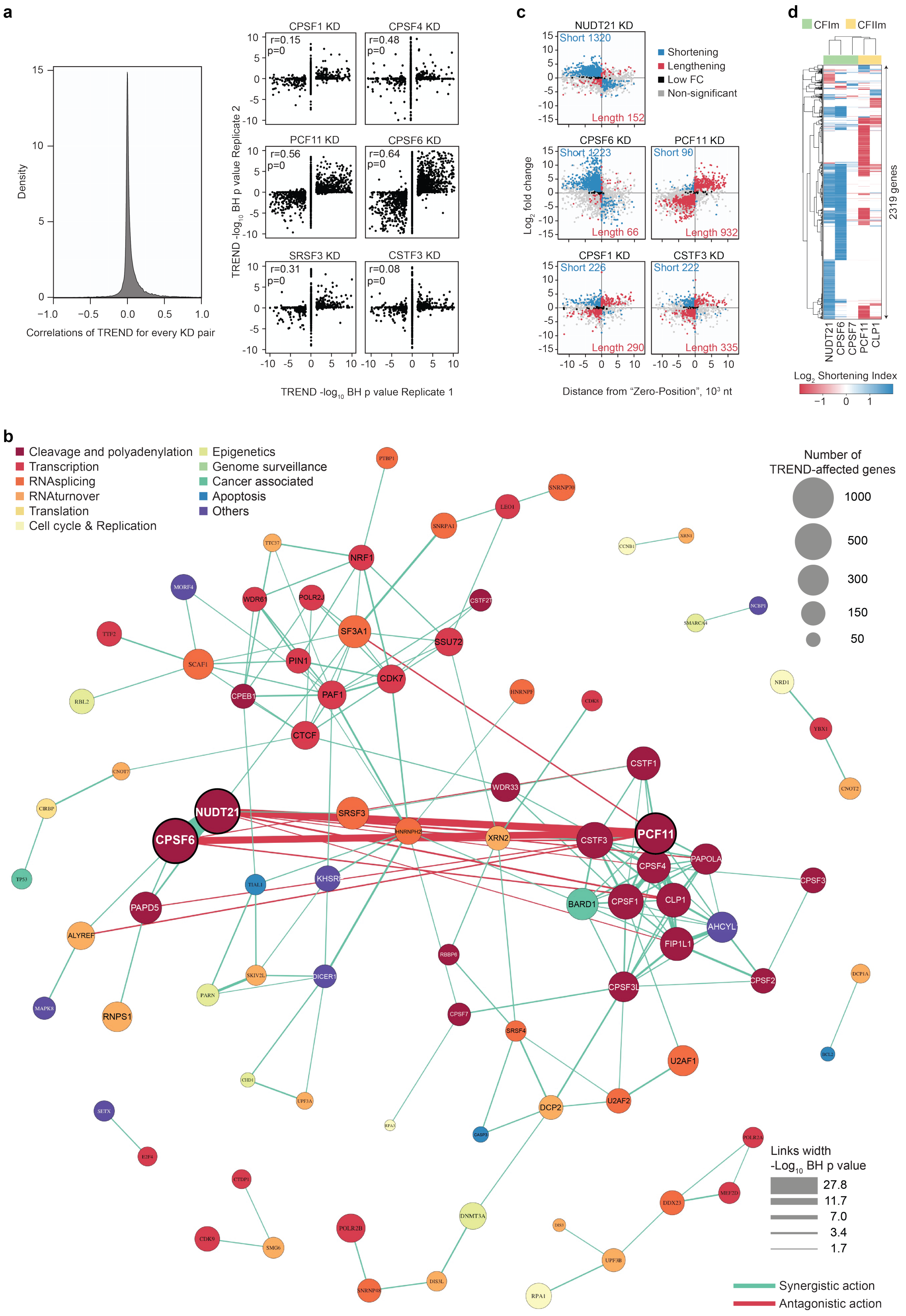

Global TREND-patterns uncover central regulatory hubs and reveal synergistic and antagonistic TREND-regulation.

a High technical and biological reproducibility of TRENDseq replicates. Pearson's correlation (*r* coefficient and *t*-test p-value) of TREND-profiles for all pairwise permutations between 174 individual depletions showing an overall poor correlation for most of the random pairs (density plot, left panel). Correlation for two independent depletion- and sequencing replicates of TREND-regulators indicated (scatter plots on the right; Pearson's correlation for most replicates is positive and exceeds more than two standard deviations from the mean, compare to density plot). **b** TREND-network analysis ('APA-network map') illustrating cooperative (and antagonistic) interactions between TREND-regulators (affecting TREND of identical genes). The diameter of the nodes reflects the number of TREND-affected genes. The links between a pair of nodes depict synergism (green, i.e. unidirectional lengthening or shortening) or antagonism (red, i.e. reciprocal lengthening or shortening) of regulation of the common pool of genes (being affected and shared upon depletion of the respective TREND-regulators). The width indicates the BH-adjusted p-value (Fisher's exact test) reflecting overrepresentation of genes being synergistically or antagonistically regulated (further details see Methods). **c** Effects of TREND on individual transcript isoforms for top 5 TREND-regulators among 174 depletions (KD=knockdown). Each dot represents a transcript isoform and the corresponding fold change (y-axis) relative to the position of the longest annotated (and significantly TREND-regulated) transcript isoform per gene ("zero-position", x-axis, for further definition see Methods). For example, PCF11-depletion up-modulates a significant proportion of transcript isoforms with 3'ends exceeding the annotated gene length (red dots in the upper right quadrant). **d** Heat map of TREND-regulation for CFIm and CFIIm complexes illustrating an overall reciprocal regulation (i.e. lengthening (red) versus shortening (blue) phenotype) with partially overlapping clusters reflecting antagonistic effects on TREND for a substantial number of identical target genes.

Supplementary Figure 4

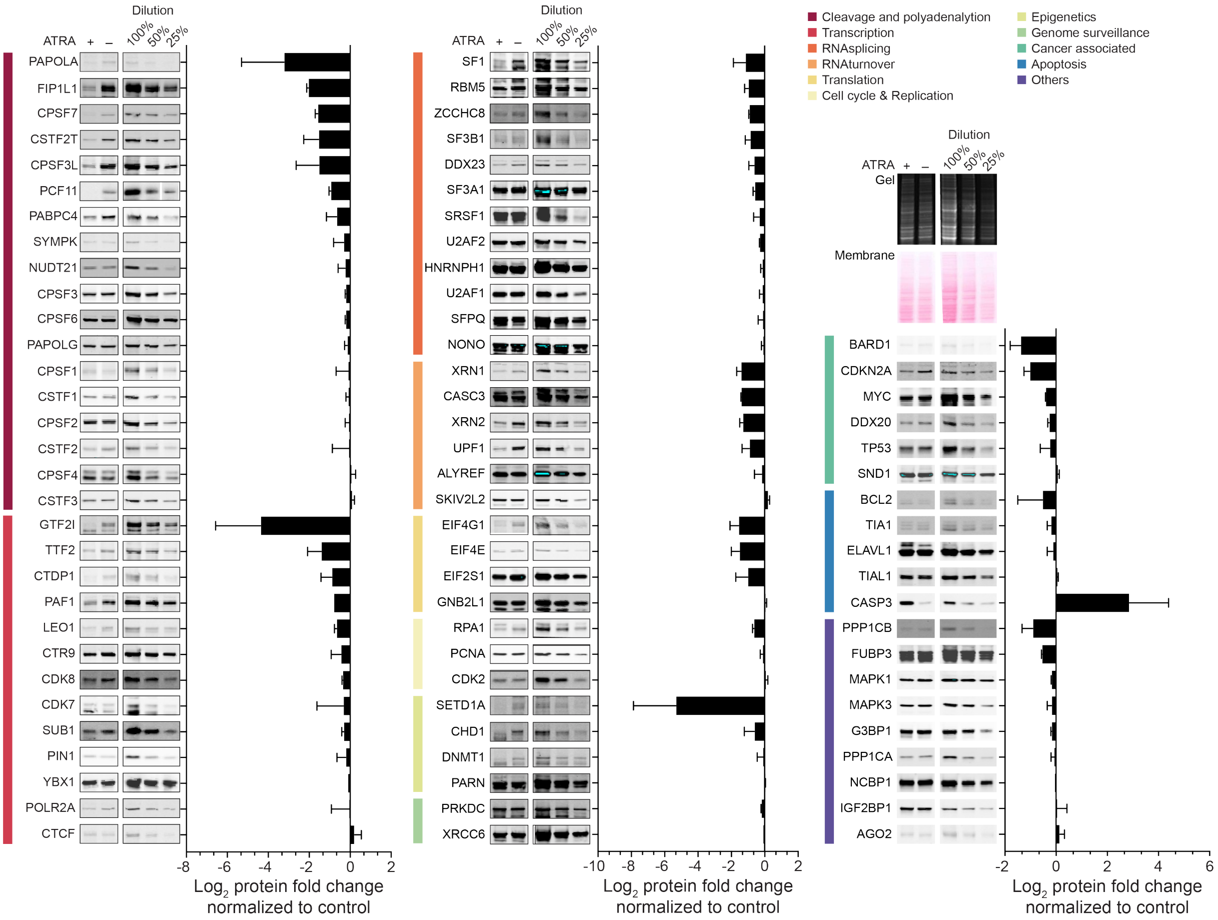

Protein profiling revealing protein abundance changes of TREND regulators during neuronal differentiation.
Total BE(2)-C protein lysate after 7 days of ATRA differentiation analysed by western blotting (equal loading is represented by in-gel and Ponceau S staining on the membrane, top panel on the right). A merge integrating the fold-regulation of protein abundance and global effect on TREND is depicted in **Fig. 3a** (error bars show s.e.m. for 2 independent replicates).

Supplementary Figure 5

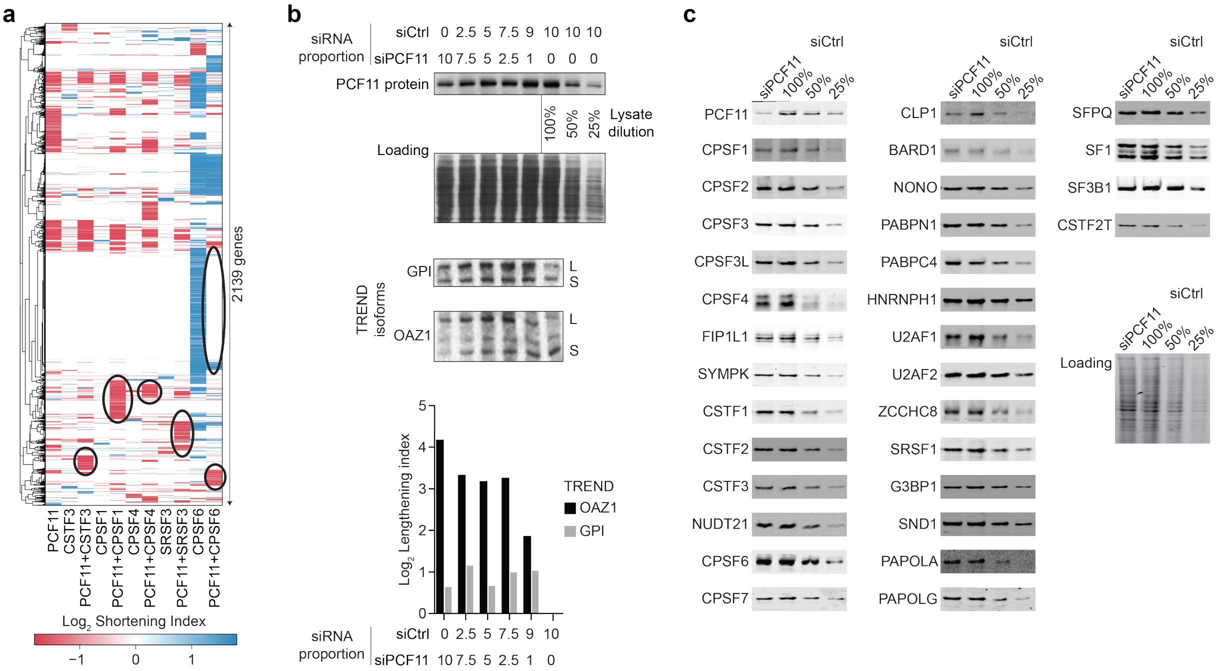

PCF11 is a key driver of TREND in neuroblastoma.

a Depletion of top TREND-regulators alone and in combination with PCF11 reveals a key role for PCF11 in the hierarchy regulating TREND. The heat map reflecting TREND-changes per gene shows substantial TREND-lengthening (red) and/or abrogation of TREND-shortening (blue; 'native' CPSF6-phenotype) upon co-depletion of PCF11. Of note, big clusters of genes showing TREND-lengthening are unique to dual depletions (highlighted) indicating that 3'elongated transcript isoforms (upon PCF11-depletion) harbour cis-elements responsive to the depletion of the co-depleted processing factor. This 'TREND-facilitating' role of PCF11 may reflect its known function in RNA Pol II pausing and/or termination control[2-4] thereby regulating the exposition of weak or strong polyadenylation signals to the trans-acting 3'end processing machinery (see discussion). **b** Minimal PCF11-alterations affect TREND most significantly (reflected by substantial lengthening of representative indicator transcripts OAZ1 and GPI, compare quantity of long (L) and short (S) transcript isoforms in northern blotting and lengthening index depicted in the lower panel; reduction of the specific PCF11 siRNA concentration by dilution down to 1:9, specific versus unspecific control siRNA, respectively, is shown in the lanes 1-5). **c** Lack of protein abundance changes of other core 3'end processing components upon PCF11-depletion suggest a direct TREND-regulation in neuroblastoma via PCF11 (representative loading control is shown in the lower right panel).

Supplementary Figure 6

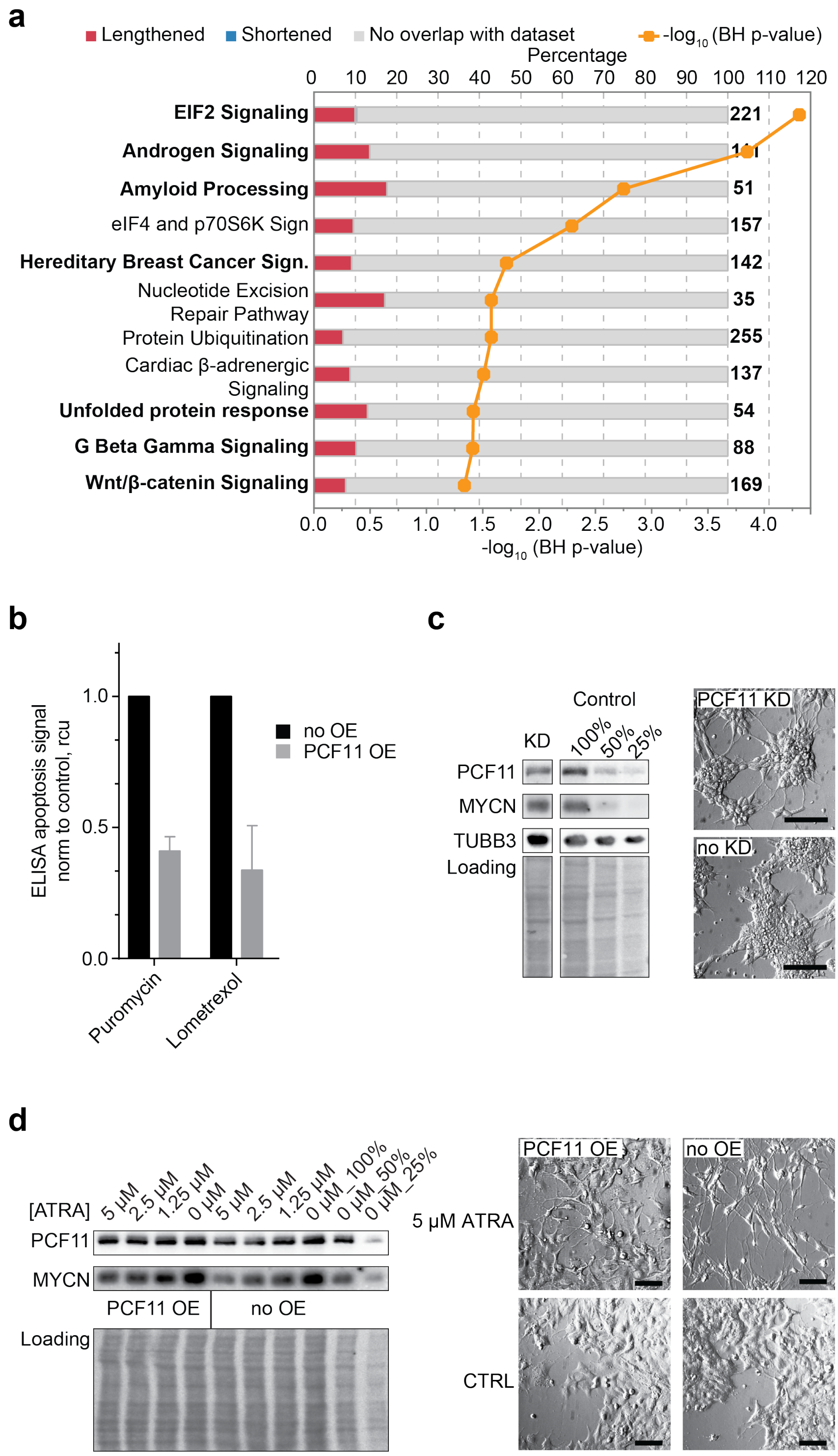

PCF11 regulates critical programs impinging on WNT linking TREND to tumorigenesis and neurodifferentiation.

a PCF11-mediated TREND-regulation significantly affects various signalling pathways including WNT (Ingenuity Pathway Analysis, BH-adjusted Fisher's Exact test p-value<0.05). Highlighted are pathways involved in tumorigenesis and neurodifferentiation (647 TREND-affected genes in at least 3 out of 5 independent PCF11-depletion replicates, **Supplementary Table 4**). **b** ELISA-DNA fragmentation assay showing that constitutive PCF11-overexpression (OE) increases resistance towards pro-apoptotic drugs such as

puromycin (n = 3) and lometrexol (n = 2) (right panel, error bars show s.e.m. for replicates). **c** PCF11-depletion in a complementary CHP-134 neuroblastoma model leads to down-regulation of MYCN and up-regulation of TUBB3 (left) and results in neurodifferentiation (micrographs on the right, scale bar 100 µm; see also main Figures e.g. Fig. 4). **d** PCF11 overexpression (OE, stably expressing cell line) antagonizes ATRA induced neurodifferentiation (i.e. MYCN down-regulation on the left and inhibited morphological changes on the right; micrographs scale bar 100 µm).

Supplementary Figure 7

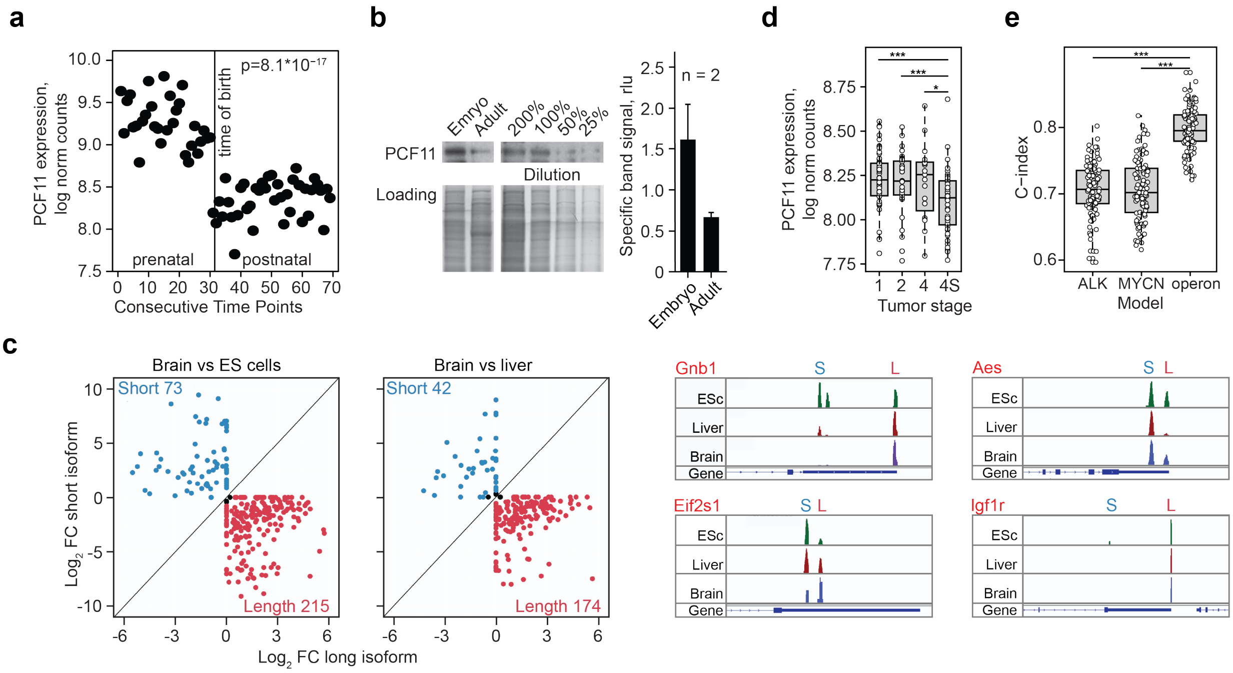

Down-regulation of PCF11 during brain development is associated with a TREND-lengthening phenotype *in vivo* and spontaneous tumour regression in neuroblastoma.

a PCF11 mRNA expression in prenatal human brain samples (n=31) is significantly higher than in postnatal samples (n=38; two-sided *t*-test). **b** PCF11 protein abundance in murine embryos and adult mice (error bars show s.e.m.). **c** Global (unidirectional) TREND-lengthening phenotype in murine brain compared to murine embryonic stem cells or liver (scatter plots on the left). Shortening and lengthening of representative PCF11-derived TREND-operon transcripts in murine ES cells, liver and brain (right panels; Igf1r, Aes, Eif2s1 and Gnb1, for further details see **Fig. 4a,b**). **d** PCF11 expression in tumour samples from age-matched patients at different neuroblastoma stages, indicating that low PCF11-expression is specific for spontaneously regressing neuroblastoma (stage 4S) as compared to metastatic (stage 4) or localized low risk (stage 1 or 2) tumours (gene expression obtained from GEO GSE49711; pairwise comparisons between stages were tested by a two-sided t-test with pooled standard deviations). **e** Cox modelling showing a superior prediction of the PCF11-dependent neurodifferentiation operon ('operon') for survival compared to established risk markers, MYCN and ALK, respectively (cox proportion hazard model was built with 0.7 of training data, using MYCN or ALK expression as independent variables. Alternatively, ratios of proximal-to-distal (APA) isoforms of PCF11-dependent neurodifferentiation operon" were used in multivariate Cox model. Modelling and validation was bootstrapped 100 times, C-index (concordance) for model validations were plotted. To assay statistical difference between models, two-sided t-test was used). For box plots **d** and **e** centre line depicts median, hinges show 25th and 75th percentile, whiskers depict interquartile range (IQR = 1.5).

Supplementary Figure 8

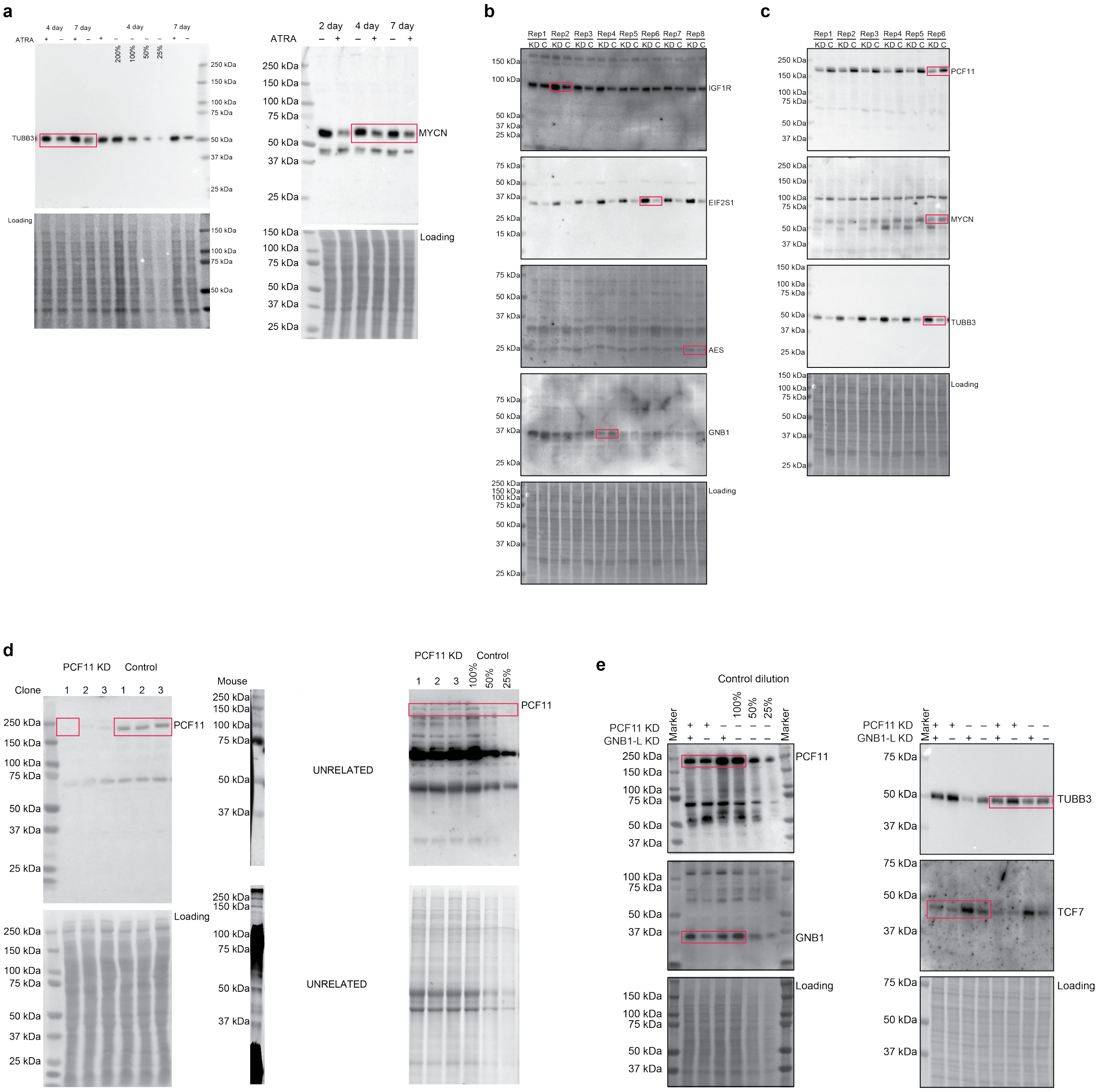

Accompanying uncropped images for main figures in the paper.
a western blots of neuroblastoma differentiation in **Fig. 1b**. **b** western blots of main regulatory hubs of signalling pathways depicted in **Fig. 4a.** **c** western blots demonstrating neuroblastoma differentiation upon PCF11 KD based on expression changes of TUBB3 and MYCN as depicted in **Fig. 4g**. **d** PCF11 expression in ES cell and brain of transgenic mouse with inducible PCF11 shRNA as depicted in **Fig. 5d**. western blots demonstrated in **Fig. 6e**. Boxed regions depict cropped parts of images presented in the **Fig. 1b, 4a, 4g, 5d, 6e**.

Supplementary Reference list

1 Zhang, Y. E., Vibranovski, M. D., Landback, P., Marais, G. A. B. & Long, M. Chromosomal Redistribution of Male-Biased Genes in Mammalian Evolution with Two Bursts of Gene Gain on the X Chromosome. *Plos Biol* **8**, e1000494, (2010).

2 Birse, C. E., Minvielle-Sebastia, L., Lee, B. A., Keller, W. & Proudfoot, N. J. Coupling termination of transcription to messenger RNA maturation in yeast. *Science* **280**, 298-301 (1998).

3 Meinhart, A. & Cramer, P. Recognition of RNA polymerase II carboxy-terminal domain by 3′-RNA-processing factors. *Nature* **430**, 223, (2004).

4 Dichtl, B. *et al.* Yhh1p/Cft1p directly links poly(A) site recognition and RNA polymerase II transcription termination. *EMBO J* **21**, 4125-4135, (2002).

Supplementary Table 1

Gene Ontology (GO) terms enrichment for TREND-affected genes upon BE(2)-C neuroblastoma differentiation triggered by ATRA. "Tandem" and "internal" TREND affect the 3'UTR and protein coding sequences, respectively.

GO_Name	-log10 p value	Type of TREND
positive regulation of hydrolase activity	3.0	Tandem
extrinsic to membrane	2.9	Tandem
endoplasmic reticulum unfolded protein response	2.6	Tandem
regulation of nuclease activity	2.4	Tandem
positive regulation of catalytic activity	2.2	Tandem
intracellular transport	2.1	Tandem
endoplasmic reticulum-Golgi intermediate compartment	2.0	Tandem
RNA polymerase II core promoter proximal region sequence-specific DNA binding transcription factor activity involved in positive regulation of transcription	2.0	Tandem
negative regulation of transforming growth factor beta receptor signaling pathway	2.0	Tandem
regulation of phospholipase activity	1.8	Tandem
secretion	2.4	Internal
establishment of localization in cell	2.3	Internal
neuropeptide signaling pathway	2.2	Internal
single-stranded RNA binding	2.0	Internal
cellular response to alcohol	1.6	Internal
negative regulation of neurological system process	1.4	Internal
DNA helicase activity	1.2	Internal
receptor-mediated endocytosis	1.1	Internal
negative regulation of cellular catabolic process	1.1	Internal
Golgi membrane	0.9	Internal

Supplementary Table 2

RNAi targets to identify key drivers controlling transcriptome 3'end diversity (TREND).

Filtered TRENDseq reads represent the reads aligned to true TREND isoforms excluding internal priming events (see Methods)

Func category	Gene Name	Number of TREND-affected genes	Mean distance between TREND regulated sites (nt)	Filtered TRENDseq reads
**Cleavage and Polyadenylation**	*CPSF1*	492	922	653905
	CPSF2	158	867	272105
	CPSF3	164	820	524221
	CPSF4	407	959	649031
	FIP1L1	399	984	596315
	CSTF1	402	1065	981763
	CSTF2	39	1141	273094
	CSTF3	536	1039	508429
	CPSF6	1163	1573	250611
	CPSF7	126	757	380527
	NUDT21	1320	1445	1169866
	PCF11	927	1573	472071
	CLP1	433	1183	673019
	CPSF3L	280	911	738161
	SYMPK	55	999	201105
	CSTF2T	115	938	587529
	WDR33	244	798	417290
	RBBP6	103	1124	243276
	CPEB1	151	1002	426169
	PAPOLA	299	1228	528244
	PAPOLG	123	1032	513028
	PAPD4	145	986	233255
	PAPD5	335	1190	1074623
	PAPD7	40	522	239409
	PABPC4	19	345	205648
	PABPN1	93	964	204929
	PABPC1	54	1506	398902
**Transcription**	*TTF2*	155	749	677614
	YBX1	114	788	370643
	CTCF	226	742	549801
	POLR2A	98	954	307657
	POLR2B	235	1033	450259
	POLR2C	87	951	364246
	POLR2E	67	811	233400
	POLR2J	164	950	468082
	GTF2I	43	1385	240473
	TBP	56	895	353003

	E2F4	84	1024	493374
	MEF2D	108	780	446531
	NRF1	165	899	490875
	PAF1	229	1091	424176
	LEO1	149	964	558264
	CTR9	43	688	195865
	CDC73	69	1140	264859
	WDR61	147	964	480531
	CDK8	98	778	403788
	CDK9	157	1057	596008
	CDK7	231	830	674539
	PIN1	157	858	602239
	SSU72	221	992	351630
	CTDP1	84	827	504107
**RNA splicing**	*U2AF1*	290	1290	593729
	U2AF2	153	1254	353909
	PTBP1	107	1196	376635
	HNRNPA2B1	128	1159	353433
	HNRNPF	155	1109	912358
	HNRNPH1	62	1050	343084
	HNRNPH2	190	1219	820272
	HNRNPH3	19	951	247690
	NONO	135	1073	825226
	SFPQ	70	794	183291
	SF3B1	30	1471	109284
	SRSF1	73	1108	236334
	SF1	111	1028	380310
	ZCCHC8	74	1097	535354
	DDX23	149	944	461848
	SF3A1	361	1181	560390
	SNRNP70	189	1444	29708
	SNRPA1	171	1318	507196
	SNRNP48	133	912	551099
	SNRPA	35	1295	215148
	SRRM1	96	1015	463756
	SRRM2	68	1071	418622
	CDC40	25	835	188326
	SRSF3	354	1277	505983
	SRSF4	100	1048	475615
	RBM5	46	669	441644
	DDX39B	67	955	255561
	SCAF1	284	974	1036661
	Nova2	32	1034	306753
**RNA turnover**	*XRN1*	55	1256	612368
	XRN2	147	795	530047
	CNOT7	71	575	547525
	TNRC6A	89	1112	389649

	SKIV2L2	22	687	367144
	TTC37	72	1084	326987
	DCP2	144	1248	613251
	UPF1	32	1630	360228
	UPF2	37	1298	396584
	UPF3A	66	1015	416470
	UPF3B	131	819	827286
	RNPS1	269	1086	790214
	ALYREF	214	1801	509470
	SMG6	103	1002	467809
	CASC3	35	832	239502
	CNOT2	111	953	569155
	DIS3	42	1113	369092
	DIS3L	91	987	636236
	EXOSC10	70	1235	428481
	SKIV2L	96	1014	611966
	ExosC6	45	1078	258519
	DCP1A	82	925	567538
	ZFP36	34	886	365198
**Translation**	*EIF2A*	28	230	234306
	EIF3C	18	701	164052
	EIF4E	45	1267	297083
	EIF4A1	103	982	605673
	EIF4G1	77	841	561227
	GNB2L1	57	1001	380891
	CIRBP	90	1133	565364
**Cell-cyle & Replication**	*RPA1*	171	1167	1084142
	RPA3	34	267	251700
	PCNA	27	1381	363853
	CDK2	64	834	368962
	NRD1	137	1071	423561
	CCNB1	67	1273	595124
	BTG2	40	368	324584
**Epigenetics**	*DNMT1*	45	1138	294528
	TRDMT1	40	938	301975
	DNMT3A	193	1149	716870
	DNMT3B	84	848	558425
	TUT1	51	903	363603
	PARN	117	1133	825793
	SLBP	56	1291	585795
	SETD1A	58	828	556751
	SETD1B	34	784	328492
	SETD2	36	843	408521
	KAT2B	49	885	340861
	SMARCA4	74	1241	641204
	CHD1	50	927	434190
	CHD2	32	802	335955

	RBL2	156	1076	772549
	SUPT16H	40	1007	386456
**Genome surveillance**	*PRKDC*	46	1220	453368
	XRCC5	27	954	396675
	XRCC6	74	900	588852
	TERT	17	1888	270488
**Cancer associated**	*BARD1*	332	1059	898038
	SND1	42	1247	331728
	MYC	75	1053	335105
	TP53	95	1024	527318
	CDKN2A	30	1084	272627
	CDKN2D	20	799	341439
	DDX20	41	757	479332
	DDX42	66	1266	265805
	XPO4	44	1242	423284
**Apoptosis**	*TIA1*	27	1210	286873
	TIAL1	99	1042	653513
	ELAVL1	31	1659	183432
	BCL2	49	1244	459697
	CASP3	57	1125	434096
**Others**	*PPP1CA*	45	468	424662
	PPP1CB	19	465	248714
	PPP1R10	77	1627	307421
	SETX	98	1146	392981
	NCBP1	75	1076	596502
	DICER1	129	902	679683
	TARBP2	103	963	736887
	AGO2	98	790	502644
	MAPK1	61	1045	544299
	MAPK3	19	832	229517
	MAPK8	121	1277	638029
	MAPK9	18	440	299948
	MAPK14	15	768	218768
	AHCYL1	295	921	594157
	JUP	50	1610	373126
	MORF4	167	817	582827
	RBM7	43	1313	363586
	IFIT1	48	966	412018
	IFIT3	58	847	587306
	NusA	12	1032	274286
	IGF2BP1	76	831	393337
	KHSRP	169	1059	496587
	FUBP3	29	1166	300709

Supplementary Table 3
List of depleted TREND-regulators and enrichment p-value of TREND-affected genes in cancer (per respective knockdown)

Enrichment analysis was performed using Hypergeometric Test on the overlap between TREND regulated and cancer associated genes (list extracted from COSMIC database http://cancer.sanger.ac.uk/cosmic).

Factor Name	Enrichment p-value	Functional category
CPSF6	1,56E-11	Cleavage and Polyadenylation
NUDT21	4,56E-10	Cleavage and Polyadenylation
PCF11	2,98E-09	Cleavage and Polyadenylation
CSTF3	8,75E-08	Cleavage and Polyadenylation
SF3A1	2,88E-07	RNA splicing
PAPD5	5,21E-07	Cleavage and Polyadenylation
PAPOLA	2,21E-06	Cleavage and Polyadenylation
CPSF4	2,28E-06	Cleavage and Polyadenylation
ALYREF	3,38E-06	RNA turnover
PAF1	3,38E-06	Transcription
CSTF1	3,64E-06	Cleavage and Polyadenylation
E2F4	7,14E-06	Transcription
HNRNPF	8,30E-06	RNA splicing
SRSF3	8,48E-06	RNA splicing
SCAF1	1,09E-05	RNA splicing
CPSF3	1,13E-05	Cleavage and Polyadenylation
CDK9	1,99E-05	Transcription
XRN1	2,06E-05	RNA turnover
HNRNPA2B1	3,81E-05	RNA splicing
ZCCHC8	4,03E-05	RNA splicing
CPSF7	4,08E-05	Cleavage and Polyadenylation
PTBP1	4,82E-05	RNA splicing
EIF4A1	6,49E-05	Translation
SETD2	6,56E-05	Epigenetics
SNRNP70	9,06E-05	RNA splicing
POLR2C	9,35E-05	Transcription
NONO	9,50E-05	RNA splicing
RNPS1	1,42E-04	RNA turnover
CPSF1	1,59E-04	Cleavage and Polyadenylation
BARD1	1,62E-04	Cancer associated
FIP1L1	1,71E-04	Cleavage and Polyadenylation
SETX	1,83E-04	Others
CLP1	1,98E-04	Cleavage and Polyadenylation
MAPK8	2,27E-04	Others
DNMT3A	2,93E-04	Epigenetics
PABPN1	3,88E-04	Cleavage and Polyadenylation
CDK7	4,50E-04	Transcription
WDR33	4,67E-04	Cleavage and Polyadenylation
PPP1CB	5,46E-04	Others
NRF1	6,07E-04	Transcription
TP53	7,12E-04	Cancer associated

U2AF2	1,04E-03	RNA splicing
AHCYL1	1,15E-03	Others
AGO2	1,16E-03	Others
LEO1	1,20E-03	Transcription
CPEB1	1,25E-03	Cleavage and Polyadenylation
SLBP	1,29E-03	Epigenetics
PAPD7	1,43E-03	Cleavage and Polyadenylation
SMG6	1,45E-03	RNA turnover
POLR2J	1,56E-03	Transcription
SND1	1,58E-03	Cancer associated
UPF3B	1,72E-03	RNA turnover
HNRNPH2	1,73E-03	RNA splicing
CPSF3L	2,03E-03	Cleavage and Polyadenylation
SFPQ	2,04E-03	RNA splicing
CTCF	2,09E-03	Transcription
U2AF1	2,16E-03	RNA splicing
SNRPA1	2,55E-03	RNA splicing
MORF4	2,86E-03	Others
EXOSC10	3,29E-03	RNA turnover
WDR61	3,32E-03	Transcription
Nova2	3,50E-03	RNA splicing
SSU72	4,17E-03	Transcription
EIF3C	4,22E-03	Translation
PAPOLG	4,67E-03	Cleavage and Polyadenylation
SNRNP48	5,30E-03	RNA splicing
TRDMT1	6,33E-03	Epigenetics
SETD1A	8,11E-03	Epigenetics
CSTF2T	8,84E-03	Cleavage and Polyadenylation
RBM7	8,87E-03	Others
POLR2E	9,16E-03	Transcription
CTR9	1,04E-02	Transcription
SRSF1	1,29E-02	RNA splicing
IFIT1	1,29E-02	Others
DDX23	1,29E-02	RNA splicing
CPSF2	1,34E-02	Cleavage and Polyadenylation
PARN	1,47E-02	Epigenetics
XRN2	1,48E-02	RNA turnover
PPP1CA	1,48E-02	Others
RBL2	1,52E-02	Epigenetics
PIN1	1,78E-02	Transcription
CDK8	1,82E-02	Transcription
CNOT7	1,83E-02	RNA turnover
XRCC6	1,92E-02	Genome surveillance
NRD1	2,08E-02	Cell-cyle & Replication
POLR2B	2,12E-02	Transcription
FUBP3	2,12E-02	Others
TTF2	2,19E-02	Transcription
SYMPK	2,27E-02	Cleavage and Polyadenylation
TBP	2,53E-02	Transcription
CTDP1	2,62E-02	Transcription

NusA	2,71E-02	Others
TARBP2	2,74E-02	Others
GNB2L1	3,12E-02	Translation
RPA1	3,12E-02	Cell-cyle & Replication
YBX1	3,14E-02	Transcription
MAPK1	3,60E-02	Others
CASC3	3,80E-02	RNA turnover
CDK2	3,95E-02	Cell-cyle & Replication
CNOT2	4,04E-02	RNA turnover
CDC73	4,32E-02	Transcription
POLR2A	4,47E-02	Transcription
SRRM1	4,63E-02	RNA splicing
PABPC4	5,26E-02	Cleavage and Polyadenylation
CCNB1	5,31E-02	Cell-cyle & Replication
RBBP6	5,63E-02	Cleavage and Polyadenylation
DDX20	5,67E-02	Cancer associated
TERT	5,74E-02	Genome surveillance
TIAL1	6,17E-02	Apoptosis
BTG2	6,28E-02	Cell-cyle & Replication
IGF2BP1	6,66E-02	Others
MEF2D	6,74E-02	Transcription
PAPD4	6,80E-02	Cleavage and Polyadenylation
SRRM2	6,90E-02	RNA splicing
BCL2	7,24E-02	Apoptosis
JUP	7,24E-02	Others
EIF4G1	7,64E-02	Translation
TUT1	7,91E-02	Epigenetics
TIA1	8,93E-02	Apoptosis
DCP1A	8,97E-02	RNA turnover
CDC40	9,51E-02	RNA splicing
CASP3	1,09E-01	Apoptosis
XRCC5	1,13E-01	Genome surveillance
UPF1	1,31E-01	RNA turnover
KHSRP	1,32E-01	Others
SRSF4	1,39E-01	RNA splicing
DDX42	1,50E-01	Cancer associated
CHD2	1,51E-01	Epigenetics
DCP2	1,66E-01	RNA turnover
SETD1B	1,70E-01	Epigenetics
SF1	1,81E-01	RNA splicing
UPF2	1,84E-01	RNA turnover
DIS3	1,90E-01	RNA turnover
TTC37	2,01E-01	RNA turnover
ExosC6	2,18E-01	RNA turnover
NCBP1	2,20E-01	Others
PRKDC	2,25E-01	Genome surveillance
SUPT16H	2,25E-01	Epigenetics
MAPK14	2,46E-01	Others
DNMT3B	2,55E-01	Epigenetics
RBM5	2,59E-01	RNA splicing

TNRC6A	2,60E-01	RNA turnover
CHD1	2,80E-01	Epigenetics
CIRBP	2,80E-01	Translation
PABPC1	2,94E-01	Cleavage and Polyadenylation
DIS3L	2,95E-01	RNA turnover
HNRNPH3	3,00E-01	RNA splicing
HNRNPH1	3,42E-01	RNA splicing
IFIT3	3,48E-01	Others
CDKN2D	3,51E-01	Cancer associated
DDX39B	3,62E-01	RNA splicing
UPF3A	4,21E-01	RNA turnover
EIF2A	4,31E-01	Translation
PCNA	4,42E-01	Cell-cyle & Replication
CDKN2A	4,52E-01	Cancer associated
ELAVL1	4,52E-01	Apoptosis
RPA3	4,82E-01	Cell-cyle & Replication
ZFP36	5,01E-01	RNA turnover
SNRPA	5,20E-01	RNA splicing
DICER1	5,27E-01	Others
CSTF2	5,55E-01	Cleavage and Polyadenylation
DNMT1	5,87E-01	Epigenetics
GTF2I	5,87E-01	Transcription
EIF4E	5,95E-01	Translation
SKIV2L	6,20E-01	RNA turnover
XPO4	6,58E-01	Cancer associated
MYC	7,74E-01	Cancer associated
PPP1R10	7,74E-01	Others
SMARCA4	7,78E-01	Epigenetics
KAT2B	1,00E+00	Epigenetics
MAPK3	1,00E+00	Others
MAPK9	1,00E+00	Others
SF3B1	1,00E+00	RNA splicing
SKIV2L2	1,00E+00	RNA turnover

Supplementary Table 4

Set of genes significantly affected by TREND (BH p ≤ 0.05 in at least 3 out of 5 PCF11 knockdown experiments)

Average Shortening Index (see Methods) with standard error of the mean (SEM) was calculated based on at least 3 independent PCF11 knockdown experiments. Color-highligted genes resemble a 'TREND-operon' (54 genes), which show a change in protein abundance (revealed by mass spectrometry, $p \leq 0.05$, $n = 3$)

Gene_ID	Average Shortening index (3 ≤ n ≤ 5)	SEM (3 ≤ n ≤ 5)
PGM3	-5,909147593	2,178837643
SLIT3	-5,435068571	1,193620518
SQSTM1	-4,11150367	0,109803159
UBE2Z	-3,826064693	0,246822948
HNRNPA3	-3,733702262	0,722401608
JMJD6	-3,403114645	0,484550147
AKT2	-3,271473985	0,388806703
SREK1	-3,247622353	0,48449496
IGF1R	-2,979687817	1,305285182
GFER	-2,947530863	0,231447769
MTHFD1	-2,87825265	0,364297259
GNG2	-2,849832262	0,783467601
MLEC	-2,833625103	0,440815426
ERO1L	-2,831014967	0,488344197
ATIC	-2,802197974	0,202541893
ELP2	-2,763840379	1,154260401
EIF2A	-2,706082257	0,361794634
IARS2	-2,652093871	0,414630251
PNN	-2,620833077	0,143100021
SRSF5	-2,609095669	0,365417587
TPM3	-2,534161935	0,75232365
RBM17	-2,507878409	0,269963842
BACE1	-2,396279323	0,761175377
RNF114	-2,344641073	0,297245414
UNC5C	-2,291310631	1,367783791
MKI67	-2,278481248	0,731171893
CUL4A	-2,230425696	0,564499893
PPP6R3	-2,228990408	0,281011098
CD2BP2	-2,061101909	0,504778102
PDXK	-2,060683308	0,303711391
TM2D2	-1,990759356	0,393485579
PVRL1	-1,961075888	0,789082738
PLEKHA6	-1,928232941	0,564233023
ASB6	-1,79048985	0,49742751
PCMT1	-1,740670755	0,253731353
MCM4	-1,734731145	0,305587889
AES	-1,670863284	0,248140822
LETM1	-1,649767036	0,287532985
ATP6V1B2	-1,591220398	0,11892995

NGRN	-1,567181868	0,341923652
EIF2S1	-1,564574129	0,147187178
NMT1	-1,562447749	0,296816244
ETF1	-1,499459853	0,199327793
PPIA	-1,45527385	0,301517695
SEC11A	-1,445460584	0,365892175
GNB1	-1,431971299	0,240385225
TM9SF3	-1,394363531	0,319841863
VPS33A	-1,375885086	0,540538905
VPS35	-1,323906194	0,407927262
TIPRL	-1,290350977	0,168814949
HNRNPDL	-1,236603365	0,495848557
HNRNPH3	-1,119833988	0,268543179
METAP2	-0,913490619	0,144926068
FDFT1	-0,57648003	0,423510183
PCYT1A	6,869721157	0,841556575
PAK1IP1	-6,829096938	0,371661133
SH3GLB1	-6,783183285	1,674836956
GUSBP11	-5,930588561	2,064389787
ZNF423	-5,857052305	0,848392584
MRPL17	-5,837012565	0,803147009
BRWD1	-5,405360901	1,756245644
AP1AR	-5,346439294	0,638288693
FAF1	-5,251668848	0,564348691
HIST2H2BC	-5,237176114	1,460600831
THAP2	-4,966272432	2,138417192
NSUN3	-4,915605533	1,594181877
SSTR2	-4,846560912	2,576424539
POLR2F	-4,812371115	0,771497962
ADARB1	-4,809457979	2,104708845
GALK1	-4,642289862	1,795479553
ARL3	-4,580642403	0,466711446
TARDBP	-4,537730589	2,674268078
FLYWCH2	-4,518596988	0,675088045
NLN	-4,463915025	1,879079125
PRPF18	-4,440941391	0,302278719
HMGN3	-4,396305701	1,331660162
ABCF3	-4,386191776	1,477182862
ZNF280D	-4,357020849	1,889296323
ZNF22	-4,34511567	0,18533431
OGT	-4,225204199	1,770970567
ESYT2	-4,216942717	1,581357517
CDS2	-4,15061157	0,534797594
RPS15	-4,085282587	0,336242357
ALG13	-4,055717878	1,474364305
SHC3	-4,045915854	1,972827072
ABI2	-4,043911022	1,544689227

DLK1	-4,034658175	0,466130526
TSPAN9	-3,988638185	1,964300665
ASB1	-3,967694197	1,659358549
HAND2-AS1	-3,965454447	0,92141727
TLX2	-3,965227091	1,06341495
TYW3	-3,940344753	1,675711936
FBXO9	-3,939901106	0,599183175
MPZL1	-3,894857439	1,595120567
EIF3H	-3,854229601	1,151338663
C11orf84	-3,7393882	0,421812365
ANKRD9	-3,724582597	0,842127236
TRAPPC3	-3,680173404	1,102532526
TTC7A	-3,630075576	0,761857599
RNPC3	-3,605551619	0,694236853
RNF44	-3,602509737	1,571810088
RAD1	-3,594632122	1,306604617
SEPT5	-3,582751167	0,706558898
NCS1	-3,575048436	1,595690253
YAE1D1	-3,569536373	0,258055898
EXOSC3	-3,558454094	0,363413629
GPN2	-3,548802793	0,835064477
PPAN-P2RY11	-3,540305152	0,436528016
RAB22A	-3,540109004	0,811906773
ITGB5	-3,534012106	0,415164304
RYK	-3,524198038	0,482736449
MAPK8IP2	-3,522764378	0,77609401
CPSF6	-3,513345539	0,632884634
PSMD9	-3,438975167	0,299229462
DYNC1H1	-3,436579071	0,329878418
HP1BP3	-3,429148085	1,171905994
ITGA1	3,426373307	1,327477192
MXD4	-3,419178982	0,339277041
SLC30A5	-3,409746475	1,537203596
FAM104B	-3,392999292	0,495296034
TMEM38A	-3,387860524	0,481275308
FAM50B	-3,366173176	0,73282894
CTU1	-3,365685231	0,436513758
SARS	3,349119713	0,668794055
GLO1	-3,337020205	1,060763976
SIGMAR1	-3,333721803	0,363379097
SPIN3	-3,316051197	0,285450126
HDAC5	-3,31140753	0,423029381
WHSC1	-3,309583343	1,132441404
TRIM4	-3,308290592	0,601622192
EIF1	-3,304002091	0,634138259
NDRG3	-3,302305304	0,352635092
AP5M1	-3,299403814	0,703955435

BCAT1	-3,291291676	0,84117016
C1orf86	-3,287849879	0,861661445
AGPAT6	-3,2702179	0,751273126
PCLO	-3,262033839	0,524771827
DNAJA2	-3,259669587	1,145806027
PDHA1	-3,251742378	1,021078428
RELN	-3,245113351	0,988991165
MKLN1	-3,244660579	1,592898488
MAP7	-3,230980099	0,4016482
MRPL35	-3,227830545	0,765809627
PPP2R5C	-3,217372774	1,611393359
FAM49B	-3,215087758	2,538655254
SENP2	-3,202095286	0,840902391
SLC25A46	-3,199275584	0,374144424
CDK5R2	-3,192088022	0,482815239
EEF1E1	3,186723572	2,438584854
MRPS11	-3,163537622	0,397583725
POMT2	-3,153313071	0,577595568
SEC61A1	-3,148703772	0,706111425
BMS1	-3,141986587	0,240316341
EIF2S2	-3,11465718	0,402647316
ENDOV	-3,112583395	0,300184616
TRABD	-3,108650437	0,609818674
CYCS	-3,10267286	0,446634456
LOC148413	-3,093644511	0,393630428
CCDC50	-3,083592792	0,473714702
FUCA2	-3,070895397	0,508310495
RAB27A	-3,052588551	0,799213309
IFT22	-3,0418927	0,06099575
KLHL7	-3,040933116	0,941615628
PTEN	-3,017904951	0,531157265
RRP15	-3,017589507	0,574870148
HMX1	-3,008431333	0,487181186
NDUFA2	-2,996494859	0,175679927
GABPB1	-2,98913258	0,468674238
DPYSL3	-2,98289896	0,431680206
FBXW8	-2,971974849	1,609619211
WDFY3-AS2	-2,971015807	1,001642712
LRP3	-2,96714579	0,566128873
H3F3B	-2,964294347	0,329607831
SNX1	-2,947377871	0,504844048
H2AFJ	-2,945211763	0,1056612
SYNGR1	-2,943979167	0,378608576
NUDT5	-2,941176048	0,425328661
COA1	-2,917397129	0,707228622
MAD2L1	-2,914683102	0,869880522
NKAP	-2,90243363	0,587111514

COX7B	-2,9020629	0,172363465
TCF3	-2,901893904	0,392433756
NR2C1	-2,897924759	0,598524879
TMED2	-2,893080186	0,354337656
C6orf89	-2,888158294	0,372725733
UBA52	-2,879348904	0,447053467
PDCD2	-2,871876462	0,726210168
SSR1	-2,867842148	0,619475944
STRAP	-2,862785747	0,477338461
DNAJC18	-2,862199973	0,579847676
CCNB1IP1	-2,85807186	0,480928371
FKBP1A	-2,848327343	0,889910698
HSPBP1	-2,840631049	0,549817437
GGH	-2,82702715	1,320338741
HSPA4	-2,818520775	0,431679679
FLJ44635	-2,818463892	1,32092399
TMCO1	-2,807184458	0,370979894
PCNX	-2,802457078	0,750355553
DCTN5	-2,798837469	1,098952981
ADAM12	-2,783051153	0,818519588
STK11	-2,780593927	0,166952412
DNAJB1	-2,767583179	0,823920169
QDPR	-2,766149929	0,319115513
MAPRE1	-2,763226145	0,567376256
HDLBP	-2,761715089	0,551951914
MAP4	-2,756429731	0,228962847
CD164	-2,751285415	1,489861974
CAND1	-2,747352982	0,434142855
ERCC2	-2,742261148	0,282132366
SRRM3	-2,737151472	0,234975192
TMEM108	-2,736743468	0,47811651
MELK	-2,735726845	0,708015104
NDUFAF4	-2,712577859	0,223269165
ABHD2	-2,712049182	0,485022875
C16orf72	-2,710928206	0,960958512
OAZ1	-2,70190943	0,275663019
C17orf75	-2,699142996	0,367504327
JAKMIP2	-2,687015798	0,127171153
PHF10	-2,676178224	0,353336534
NAT8L	-2,671986797	0,542562785
RIMBP2	-2,671680686	0,752918981
PCCB	-2,664098231	0,803129332
ASPHD1	-2,661903562	0,422902473
DPH3	-2,65096563	0,192183671
TPM1	-2,650046384	1,53661033
CLCN7	-2,645606604	0,440520233
C1orf21	-2,641522777	0,554274346

TOR1A	-2,639631026	0,322225714
NUP160	-2,638987862	1,990441557
KDELR2	-2,632061819	0,260342309
ASIC1	-2,627891134	0,434632093
SCAF4	-2,626518951	0,399326131
TMEM57	-2,622524161	0,283577103
CENPA	-2,613622502	0,229733879
USP9X	-2,612132733	0,465630239
RFC3	-2,607545138	0,282386406
MAN2A2	-2,605067755	0,627754827
FAIM2	-2,589231966	0,468237011
NOL10	-2,588494632	0,208531979
RPL27A	-2,577431732	0,3879488
ATG12	-2,577258094	0,317854088
STC2	-2,576882782	0,578209581
REEP5	-2,575117565	0,321803018
SCFD2	-2,572355081	0,558682033
BOD1	-2,571539721	0,433311217
PARD3	-2,562903568	0,526945229
RPS23	-2,561960288	0,479669518
MEF2D	-2,559775963	0,694567313
NPLOC4	-2,545639146	0,55592158
KTN1	-2,543047625	0,912119544
NSL1	-2,54059836	0,488203955
RNFT2	-2,525000159	0,370769327
UBE2D2	-2,521979694	1,208188711
ICMT	-2,515931129	0,223945625
KCMF1	-2,514532202	0,336867677
GFM1	-2,504845813	0,661177513
MDH2	-2,497682104	0,89714787
RARA	-2,491207484	0,450014486
PPHLN1	-2,491114519	0,124001104
MIR4697HG	-2,485280248	0,28349366
CBX5	-2,481791844	0,811115891
PRKRIP1	-2,476415283	0,427911055
SEPT6	-2,4725335	0,611924543
U2SURP	-2,466476901	0,087633372
TMED10	-2,459978649	0,316538874
REEP3	-2,45877555	0,416695889
ERCC1	-2,44930494	0,558038391
CACNG4	-2,436554135	0,427831422
SRP19	-2,43327068	0,464357088
OTUD5	-2,43233178	0,478148169
ABCB1	-2,430438013	0,250995073
PURB	-2,425307748	0,35192812
ST8SIA3	-2,423813289	0,416512872
UBA3	-2,419692268	0,709861421

DOT1L	-2,411235563	0,736786007
SAR1B	-2,406239543	0,36927981
MRPS23	-2,403648613	0,357681883
MARCH6	-2,395457237	0,823387728
TSPAN3	-2,395304963	0,68189497
ABLIM1	-2,393590036	0,475759369
MGAT5B	-2,389511599	0,451122485
GPT2	-2,388135086	0,443711317
SEC14L1	-2,372298999	0,789601574
UVRAG	-2,371673619	0,246560333
FAR2	-2,36738628	0,356368754
CHORDC1	-2,363998993	0,501292351
THY1	-2,361221751	1,05305756
CACUL1	-2,359574345	0,250496866
ENAH	-2,357541672	0,451267155
HAGHL	-2,355855406	0,394237549
SCARB2	-2,348067902	0,344296898
AK2	-2,34752478	0,22039571
CCND1	-2,347355903	0,363640149
ACSL3	-2,342092191	0,640448774
DLGAP4	-2,33708416	0,03579145
SNX13	-2,335306992	0,46086325
DZIP3	-2,330723728	0,227239932
IGFBPL1	-2,316685398	0,338657269
PHLDA1	-2,315776658	0,32900941
PPP6C	-2,314484125	0,423829544
TMEM237	-2,309235618	0,324533673
NARF	-2,304650996	0,176479657
COL4A3BP	-2,299578407	0,07990677
DUSP4	-2,29759679	0,484100834
TMBIM6	-2,294180047	0,48968045
MAGI2-AS3	-2,294046909	0,410962098
WHSC1L1	-2,294037749	0,459894359
RNASEH1	-2,287989964	0,26519552
HSPA5	-2,283946243	0,424087137
HNRNPD	-2,277990053	0,379395572
DCAF8	-2,274897617	0,31520591
RALA	-2,27433347	0,362800242
MCM7	-2,273283519	0,574806892
USP7	-2,271419668	0,255084923
PPP2R5E	-2,270193349	0,307272947
SC5D	-2,267661238	0,606207765
EXOC7	-2,265159677	0,256862886
CTDSPL2	-2,25983209	0,937718641
FAM208A	-2,25702043	0,199706496
DLD	-2,25617422	0,43775174
ZC3HAV1	-2,251103787	0,565955923

NUDT21	-2,245841772	0,55166502
TP53BP1	-2,240936806	0,270014526
HIST1H2BD	-2,236015979	0,899777552
C3orf14	-2,230492728	0,292244525
OXCT1	-2,224366112	0,42345329
TM9SF1	2,207123834	0,450714922
VAPB	-2,201363471	0,145845473
SEC63	-2,201291137	0,480944961
MBOAT2	-2,200887386	0,420805265
ITFG1	-2,194714516	0,711553603
AP1S2	-2,194599525	0,287005997
CCDC71L	-2,192515539	0,448274324
WDR5	-2,190563154	0,768476739
GRHPR	-2,187557753	0,83554446
RAD50	-2,179921992	0,413522578
CABLES1	-2,175091653	0,47779168
ELP3	-2,164469743	0,278620413
TMEM123	-2,158736901	0,201621805
SUV420H1	-2,14643584	0,439144466
RYBP	-2,142984424	0,385429643
SCO1	-2,142642491	0,206270959
COPZ1	-2,140684719	0,408469379
TBL1XR1	-2,136918131	0,822434684
PTN	2,134660379	0,191369532
RPS28	-2,133652001	0,402387852
GSPT1	-2,127647481	0,278694241
POLR2E	-2,12451148	0,302114247
C1orf43	-2,124346466	0,24753785
GNG4	-2,115045965	0,533961321
UBE2L3	-2,11409432	0,278266141
SIKE1	-2,113912404	0,393684245
PGK1	-2,11106203	0,538289445
CBX6	-2,110858066	0,573006365
ATP2A2	-2,109730273	0,341645
WNK1	-2,103144129	0,340045621
LSM4	-2,103104346	0,237499692
SEPT8	-2,096827103	0,460731901
RGS4	-2,092575486	0,316839662
TCEB2	-2,090765014	0,397474192
HN1	-2,084484659	0,270650518
CALU	-2,082245932	0,284876987
TMEM14A	-2,075722054	0,287145618
TMA7	-2,073614029	0,261600471
FUBP1	-2,068296151	0,284323236
BNIP2	-2,067801981	0,347327893
POLR2K	-2,066473955	0,323950467
GAS8	-2,06553216	0,236924497

TROVE2	-2,064243252	0,165761823
DHX8	-2,062879695	0,263872131
CNOT7	-2,061331852	0,104047298
BCCIP	-2,060116203	0,450918047
HUWE1	-2,046211814	0,214887682
MINOS1	-2,046193108	0,433036536
XPOT	-2,045570845	0,545321664
URM1	-2,042003026	0,35041033
RBBP6	-2,041375335	0,723330826
SRPRB	-2,041215936	0,423577182
NAV1	-2,034699422	0,383980046
NRP1	-2,029274064	0,368729041
LPPR5	-2,017806815	0,406200767
SURF6	-2,017670324	0,177510443
CDK16	-2,012710912	0,535614866
YIPF6	-2,010647067	0,917293514
MTHFD1L	-2,009883183	0,572274958
TMEM167A	-2,005192927	0,993004605
RERE	-2,003545799	0,627603004
SRI	-1,995141291	0,235448074
CBX8	-1,993022668	0,530329497
DCAF7	-1,991986012	0,414687926
MSL1	-1,980560039	0,096009931
RER1	-1,975782364	0,471197905
GPI	-1,974898734	0,331862483
MCFD2	-1,973779022	0,206810067
INTS1	-1,973318588	0,287570692
SAR1A	-1,97175011	0,361721909
SRSF6	-1,967886062	0,770692051
MRPL42	-1,966700541	0,396053787
CCDC25	-1,959937289	0,562836572
DDX5	-1,959934579	0,244137358
UBE2I	-1,959744819	0,417691211
FTH1	-1,957248239	0,574198672
SSU72	-1,956890567	0,448344624
DOK4	-1,95421542	0,573861321
TBRG1	-1,950841245	0,406650434
CLSTN2	-1,947515837	0,862661041
DRAM2	-1,937648081	0,233446108
CSNK1D	-1,931223346	0,375954453
TRIP12	-1,928502235	0,353255958
PSMD12	-1,918035448	0,397599282
RAB11A	-1,912999378	0,776255308
MKKS	-1,903247095	0,683462257
EIF1AX	-1,902502539	0,118526293
C17orf80	-1,901648601	0,419034772
ERP44	-1,88745698	0,5001642

PACS2	-1,885654935	0,278878856
GOLGB1	-1,882726738	0,264587789
ITM2B	-1,874860875	0,218631617
DHX15	-1,873356661	0,224742429
MMAB	-1,872776466	0,262095984
TPP2	-1,871954837	0,449244538
CCT5	-1,870977381	0,45082496
GNAO1	-1,869202508	0,39423626
KIF2A	-1,866464546	0,525428133
CENPN	-1,865334142	0,636650287
MRPL3	-1,863071563	0,479801523
SEPT2	-1,856930572	0,161383783
ADRBK2	-1,855873341	0,378192945
CD47	-1,853379293	0,227439726
SCAMP2	-1,852583213	0,156363685
CRYGS	-1,847437921	0,57019433
MAP2	-1,841043809	0,312702873
CLPB	-1,837225114	1,170272741
TRAF7	-1,829177643	0,217448808
TOMM20	-1,824035446	0,932542825
ZWILCH	-1,81560518	0,93877314
METTL21A	-1,813865495	0,131890636
DIDO1	-1,809698437	0,508326477
BRIX1	-1,805088767	0,275766376
COMMD2	-1,796688196	0,535138414
HLTF	-1,789084994	0,212397316
GRB2	-1,787030639	0,207790133
GSK3B	-1,771844136	0,39564148
RPS6KA6	-1,771458102	0,489190508
METTL9	-1,76890454	0,321272882
CARHSP1	-1,765318115	0,268061667
ANKHD1	-1,761915227	0,677814248
NXPE3	-1,760575913	0,819586239
RPL28	-1,755968866	0,153209789
MGEA5	-1,752782147	0,475759476
AGFG1	-1,750393232	0,341828872
NDUFC2	-1,747640285	0,398578789
FKBP3	-1,73700099	0,42669827
CSNK2A1	-1,724380289	0,891404803
PSMG4	-1,723080383	0,199222053
MCM8	-1,722068267	0,965938028
CBR4	-1,718279549	0,197192034
CAMTA1	-1,718036432	0,760609586
CNIH4	-1,716986325	0,192287591
NUP155	-1,702791544	0,436096443
RBM33	-1,698954373	0,426892233
TMEM248	-1,693554531	0,302773647

TM9SF4	-1,692105012	0,280429805
LUC7L3	-1,673445035	1,192833016
AKAP8	-1,673309934	0,211999845
RB1CC1	-1,670691089	0,256731333
HSPE1	-1,667935096	0,534869276
AGTPBP1	-1,665495188	0,572054518
NEAT1	1,650512772	1,449789123
MTA3	-1,648009432	0,447032243
CYB561	-1,64743469	0,149202149
QPRT	-1,641488292	0,329258293
AF079515	-1,63732657	0,252377322
UCK2	-1,631759222	0,193729283
CNIH1	-1,626917742	0,276164034
PDS5A	-1,625259215	0,774256617
UBE2K	-1,625054247	0,287605487
DDX56	-1,624215381	0,195514971
TRAK2	-1,621790074	0,518319758
MRRF	-1,618697559	0,399428385
BAALC	-1,614132907	0,292756755
ARL6	-1,613756237	0,123873214
HNRNPA2B1	-1,613427176	0,457110978
COX11	-1,610255201	0,463753592
SNX3	-1,593170625	0,507760799
RGS5	-1,589147492	0,089750927
RSL1D1	-1,585649291	0,184184266
DERL1	-1,580287065	0,63150843
SLC38A10	-1,575632955	0,209108835
SPPL2B	-1,575364151	0,207345637
AURKA	-1,571893223	0,391340366
NDUFS3	-1,566635797	0,489305451
CNOT1	-1,563022765	0,02859942
AP2B1	-1,560919307	0,436529198
CADM1	-1,560624903	0,331019304
COX15	-1,553300449	0,20017704
FAM213A	-1,54848978	0,225609175
ATG4B	-1,547313027	0,072731442
VIP	-1,54623904	0,186527169
ASXL1	-1,541182226	0,341895605
SRSF11	-1,528427735	0,174647486
SLMO2	-1,519638705	0,401338753
GTPBP4	-1,51795013	0,257848012
HSBP1	-1,517574572	0,144718919
DYNC1LI1	-1,516767294	0,227469049
CSE1L	-1,516562403	0,227355175
NARS	-1,515536802	0,213260483
AK094990	-1,514473964	0,394057339
LARS	-1,506867237	0,20270657

CAMSAP1	-1,505480887	0,358243131
PRPF4B	-1,505237798	0,170319704
LEPROTL1	-1,503484115	0,674556404
TMEM33	-1,500066376	0,477202529
ABCE1	-1,498254189	0,401571082
TUBB3	1,489370252	0,146681586
TIAL1	-1,48616485	0,329750403
YME1L1	-1,485723202	0,357279095
C7orf73	-1,480330888	0,291597148
NAMPT	-1,478697992	0,40154345
AMOTL1	-1,475615271	0,517449242
NFASC	-1,471204738	0,470106441
SERP1	-1,45970181	0,462047763
DMXL1	-1,458448137	0,249160995
JPX	-1,454445031	0,458700669
AP3S2	-1,450885803	0,265222552
PRKAR1A	-1,446138405	0,463491948
SPAG9	-1,445658068	0,152993234
SF3B1	-1,444194821	0,245076429
MYL12A	-1,443914407	0,350027894
HN1L	-1,440547917	0,241902462
SYNCRIP	-1,439232896	0,456814823
RPL15	-1,436527018	0,268263489
FAM222A	-1,427795727	0,303961557
NEFL	-1,427735945	0,191235223
INSIG1	-1,426731151	0,384748671
ISOC1	-1,425530906	0,51987966
EIF4A2	-1,425335329	0,250362922
UBE2N	-1,422810829	0,348022607
ATP5F1	1,419750828	0,049077041
CSNK1E	-1,409733562	0,131288156
SNX5	-1,407773159	0,232858384
GOLM1	-1,40577307	0,397479742
SAP18	-1,401131287	0,269411473
ECHDC1	-1,395039304	0,153355115
EXO1	-1,391874731	0,412885917
AKAP12	-1,391858159	0,202034191
PSME3	-1,390307386	0,345941513
DAZAP1	-1,386217235	0,539844399
SRSF3	-1,386091147	0,447874329
BUB3	-1,38215554	0,363311704
WIPI2	-1,377344966	0,293846607
TRAP1	-1,364331521	0,308910174
SLC7A14	-1,360983036	0,252123219
OAZ2	-1,358457298	0,314676923
PMEPA1	-1,35281697	0,157794757
DSTN	-1,341578251	0,680601889

PCDH9	-1,32823725	0,829191153
GNA12	-1,324763423	0,326705401
SSR3	-1,32299544	0,715210888
ADNP	-1,317393602	0,403992244
HNRNPU	-1,31353213	0,481774331
CSDE1	-1,312762268	0,264615938
DKK1	-1,31074071	0,381919153
RET	-1,305645056	0,702554592
PA2G4	-1,296633229	0,050946175
NCLN	-1,294690899	0,452910744
VKORC1L1	-1,291021912	0,150517784
ANP32E	-1,284975099	0,309813081
GRSF1	-1,274766609	0,163656505
DESI1	-1,274276972	0,393933016
ARPP19	-1,268693878	0,375383196
BIRC5	-1,266293212	0,237098058
SEPHS1	-1,262620823	0,348418401
VDAC2	-1,260931153	0,302248458
RAB7A	-1,247895065	0,265384378
AGPAT5	-1,235497843	0,624587486
SLC25A32	1,222843708	0,312440582
DDC	-1,214282478	0,236497807
TFAP2B	-1,213942763	1,157844027
PAICS	-1,212540465	0,311801471
CIRBP	-1,20621385	0,108888699
DIMT1	-1,205060374	0,228172615
ARRB1	-1,20409598	0,40849572
MTCH2	-1,203496833	0,174031053
RHOA	-1,201378159	0,429752121
SNRPF	-1,160359301	0,081249885
GET4	-1,160127992	0,177985363
NDFIP1	-1,157655263	0,178395949
SREBF2	-1,148527697	0,077832335
SDC2	-1,129881933	0,299866073
C14orf2	-1,129306472	0,240243836
PRKAR2B	-1,125124133	0,182664134
IMP4	-1,120505041	0,581541826
PSMA2	-1,109250562	0,064561201
MTDH	-1,095042765	0,094982725
MYH10	-1,092744876	0,273723622
CALM3	-1,089900522	0,518931809
PPM1A	-1,065888089	0,181764032
H2AFV	-1,064372515	0,314703661
RBM25	-1,056548295	0,102088446
PPP1CC	-1,049947888	0,183490717
EIF3J	-1,047768886	0,240872548
ACLY	-1,04283167	0,395038786

STX6	1,04273034	0,252728318
VPS29	-1,038634711	0,316155676
NDFIP2	-1,030258583	0,906630525
MLX	-1,019963494	0,200578113
SMARCA4	-1,019651155	0,420946119
TMEM106B	-1,014106941	0,052501479
TCEB1	-1,01110854	0,125918389
LMAN2	-0,992226678	0,131704141
NAP1L1	-0,989271801	0,879671537
HSPA9	-0,988697574	0,180893388
RAD23B	-0,983052029	0,365658478
UBE2D3	-0,980110634	0,200795416
TUBB2B	-0,971832192	0,212019759
CCT6A	-0,963846667	0,323983959
NHP2L1	-0,960611478	0,218817588
STT3A	-0,960401984	0,088239574
STAU1	-0,955253822	0,144595947
ABCB6	0,948048204	0,23207313
ARL6IP5	-0,94661117	0,200847935
GATAD2A	-0,939111514	0,727610152
ILF3	-0,930336869	0,160907582
CALM2	-0,929493925	0,119404101
PCBP2	-0,904223209	0,187995718
JB242118	-0,903409603	1,033084025
PRELID1	-0,879487064	0,128923045
C7orf55-LUC7	-0,878941863	0,524157004
ANP32A	-0,878568955	0,315815085
GHITM	-0,875670679	0,22784753
HAND2	0,87409662	1,0129975
SUB1	-0,850718257	0,984583022
CTSB	-0,838124459	0,111480562
SCG3	-0,819750487	0,93929666
NONO	-0,731140201	1,409817038
HMGB1	-0,694024117	1,017038747
CSNK1A1	-0,66678236	0,083326402
TRA2B	-0,623947217	0,375205883
IPO5	-0,579230238	0,756642998
RPL37A	0,56503802	0,516682457
SLC6A2	-0,550164005	1,54596379
tRNA(Ile)	0,524976167	0,340847482
JB242131	0,505469838	0,153099589
NCL	-0,435125586	0,277837323
PRDX6	-0,047455048	0,644827853
RCC1	-0,034008255	1,01809827

Supplementary Table 5

TREND-affected genes and statistical significance (p-value) of difference between long vs short TREND-isoform abundance ratio in stage 4s samples (n=48) compared to stage 4 samples (n=65) (Data downloaded from GSE49710 and RMA normalized)

Pairwise Student's *t* -Test was performed to identify significant differences between cohorts

Relative proportion of number of genes affected by lengthening, shortening or not significantly affected (NS) by TREND is summarized in Figure 6b

Gene Name	p-value	long/short ratio in stage 4S - 4	Direction of regulation 4S vs 4
ADAM22	2,32E-26	0,17	Elongation
SLC7A14	8,50E-23	0,14	Elongation
RIMS3	4,67E-22	0,15	Elongation
PLEKHA6	1,11E-21	0,16	Elongation
PVRL1	1,25E-20	0,17	Elongation
KPNA4	3,94E-20	0,09	Elongation
ARL5B	1,79E-18	0,11	Elongation
GNG2	2,00E-18	0,16	Elongation
TMEM33	3,90E-18	0,05	Elongation
TSHZ2	6,46E-18	0,06	Elongation
TCF7L2	3,65E-17	0,16	Elongation
GAN	5,32E-17	0,10	Elongation
SYNE3	7,82E-17	0,13	Elongation
LUC7L3	7,82E-17	0,09	Elongation
FBXO9	1,93E-16	0,06	Elongation
EEF2K	2,69E-16	-0,07	Shortening
USP12	3,64E-16	0,09	Elongation
DTD2	5,55E-16	-0,11	Shortening
MBOAT2	1,69E-15	0,08	Elongation
DSCR3	2,77E-15	0,10	Elongation
DUSP3	1,02E-13	0,11	Elongation
C3orf14	1,02E-13	0,06	Elongation
PCYOX1	5,42E-13	0,08	Elongation
LSM11	5,42E-13	0,05	Elongation
IGF1R	8,41E-13	0,09	Elongation
GNG4	8,63E-13	0,07	Elongation
SAR1B	9,50E-13	0,05	Elongation
RBBP4	2,07E-12	0,05	Elongation
CLEC2D	2,70E-12	-0,17	Shortening
REEP3	3,18E-12	0,07	Elongation
NCOA7	8,09E-12	0,07	Elongation
GNB1	9,79E-12	0,08	Elongation
SLC30A1	1,15E-11	0,08	Elongation
ATP11B	5,15E-11	0,07	Elongation
PCGF3	5,51E-11	0,07	Elongation
GID8	5,56E-11	0,09	Elongation

UBR1	5,94E-11	0,09	Elongation
MAPRE2	8,18E-11	0,05	Elongation
DIS3	1,08E-10	0,05	Elongation
NDFIP2	1,14E-10	0,07	Elongation
ABI2	1,62E-10	0,06	Elongation
DPH3	1,87E-10	0,07	Elongation
IGSF3	2,31E-10	0,07	Elongation
ZNF226	2,31E-10	-0,06	Shortening
PIK3C3	2,89E-10	0,05	Elongation
VAPA	2,89E-10	0,04	Elongation
HS6ST3	3,56E-10	0,23	Elongation
TMEM106B	3,56E-10	0,06	Elongation
DUSP4	4,87E-10	-0,06	Shortening
PURB	6,15E-10	0,06	Elongation
ATF2	9,37E-10	0,06	Elongation
SPAST	9,55E-10	0,05	Elongation
C9orf40	1,15E-09	0,04	Elongation
SBNO1	1,26E-09	0,07	Elongation
TBL1XR1	1,53E-09	0,07	Elongation
DCAF5	1,73E-09	-0,04	Shortening
RIOK2	1,73E-09	0,03	Elongation
MIR4697HG	1,74E-09	0,05	Elongation
ALDH3A2	1,74E-09	0,03	Elongation
PDE4C	1,81E-09	0,13	Elongation
DENR	2,87E-09	0,04	Elongation
UNC5C	3,10E-09	-0,08	Shortening
EXOSC6	3,20E-09	-0,06	Shortening
CAND1	3,63E-09	-0,06	Shortening
RABGEF1	3,63E-09	0,05	Elongation
SLC6A15	3,66E-09	0,04	Elongation
GPCPD1	3,66E-09	0,05	Elongation
STXBP5	3,66E-09	0,07	Elongation
RCOR3	3,70E-09	0,05	Elongation
GSTM3	3,84E-09	0,03	Elongation
NAA30	4,42E-09	0,04	Elongation
ELK3	4,83E-09	0,08	Elongation
ZNF764	5,95E-09	-0,07	Shortening
NOLC1	7,01E-09	0,03	Elongation
PUM2	7,57E-09	0,04	Elongation
WDHD1	7,98E-09	0,04	Elongation
KIF1C	9,08E-09	-0,02	Shortening
RNF141	9,97E-09	0,07	Elongation
KLF3	1,03E-08	0,08	Elongation
GABRB3	1,05E-08	0,03	Elongation
CMKLR1	1,17E-08	0,08	Elongation
EPB41L4A-AS1	1,17E-08	0,05	Elongation
RP5-1039K5.19	1,33E-08	0,04	Elongation
PGK1	1,35E-08	-0,02	Shortening
TCF3	1,39E-08	0,06	Elongation
PPM1A	1,81E-08	0,05	Elongation

TMOD2	1,91E-08	0,05	Elongation
ESYT2	2,26E-08	0,05	Elongation
FAM49B	2,38E-08	0,06	Elongation
UTP15	2,45E-08	-0,05	Shortening
ELP2	2,63E-08	0,04	Elongation
FGFR1OP	2,64E-08	0,06	Elongation
SAR1A	2,75E-08	0,05	Elongation
SESN3	2,82E-08	0,08	Elongation
RABEP1	2,97E-08	0,07	Elongation
CD2BP2	3,39E-08	0,06	Elongation
CLSTN2	4,12E-08	0,08	Elongation
CASC10	4,32E-08	0,05	Elongation
TMCO1	4,33E-08	0,02	Elongation
ARPP19	4,38E-08	-0,04	Shortening
VPS13A	5,34E-08	0,03	Elongation
SEC63	5,39E-08	0,03	Elongation
MPZL1	5,76E-08	0,07	Elongation
CTNND1	6,80E-08	0,06	Elongation
TSN	6,89E-08	0,03	Elongation
KLHDC10	7,26E-08	0,06	Elongation
PPP1CB	7,63E-08	0,06	Elongation
LIN7A	7,86E-08	0,08	Elongation
BCDIN3D	8,51E-08	0,03	Elongation
AES	8,54E-08	0,05	Elongation
SIRT5	9,78E-08	0,04	Elongation
ATP5E	1,00E-07	0,05	Elongation
SMAD3	1,02E-07	-0,05	Shortening
UBQLN1	1,06E-07	0,04	Elongation
UBE2D3	1,18E-07	0,04	Elongation
NUDT21	1,34E-07	0,09	Elongation
SYNJ1	1,34E-07	0,06	Elongation
WIPI2	1,34E-07	-0,02	Shortening
MAPKAPK5	1,40E-07	0,05	Elongation
TRIL	1,40E-07	0,04	Elongation
DPYSL3	1,48E-07	0,12	Elongation
RYBP	1,48E-07	-0,03	Shortening
EGLN1	1,49E-07	0,05	Elongation
EFCAB14	1,66E-07	-0,07	Shortening
CPNE3	1,73E-07	0,04	Elongation
RPS6KA6	1,85E-07	0,04	Elongation
SYT11	1,99E-07	0,04	Elongation
VAMP4	2,05E-07	0,05	Elongation
ELP3	2,36E-07	0,03	Elongation
SYNRG	2,67E-07	0,07	Elongation
PGBD5	2,68E-07	0,04	Elongation
LMAN1	2,77E-07	0,04	Elongation
KLHL36	2,90E-07	0,06	Elongation
FOXC1	2,92E-07	-0,06	Shortening
AKT2	3,32E-07	0,03	Elongation
RBM18	3,52E-07	0,04	Elongation

SLC1A4	3,67E-07	0,04	Elongation
TMEM87B	4,17E-07	0,04	Elongation
NUFIP2	4,53E-07	0,04	Elongation
DCP2	4,88E-07	0,07	Elongation
ASXL1	5,90E-07	0,04	Elongation
GNB4	5,98E-07	0,06	Elongation
ZSCAN29	6,00E-07	0,04	Elongation
SMIM14	6,00E-07	-0,02	Shortening
TLDC1	6,05E-07	0,04	Elongation
SNRPB2	6,57E-07	0,03	Elongation
MED12L	6,79E-07	-0,05	Shortening
PSMD12	8,03E-07	0,04	Elongation
RBM17	8,77E-07	0,03	Elongation
PGGT1B	9,83E-07	0,05	Elongation
PTP4A1	1,01E-06	0,04	Elongation
MXRA7	1,06E-06	0,03	Elongation
TXNL1	1,06E-06	0,01	Elongation
AGFG1	1,09E-06	0,03	Elongation
FAM199X	1,13E-06	0,04	Elongation
NEK9	1,17E-06	0,06	Elongation
TMCC1	1,30E-06	0,05	Elongation
CCDC90B	1,36E-06	-0,02	Shortening
RERG	1,43E-06	0,03	Elongation
IVNS1ABP	1,63E-06	0,02	Elongation
GNA13	1,63E-06	0,06	Elongation
DYNLL2	2,03E-06	0,07	Elongation
CEP97	2,21E-06	0,03	Elongation
EIF3H	2,24E-06	-0,04	Shortening
DUSP16	2,86E-06	0,04	Elongation
TMEM108	2,95E-06	0,03	Elongation
TMTC1	2,97E-06	0,05	Elongation
PRKD3	3,64E-06	0,06	Elongation
MAD2L1	4,08E-06	0,04	Elongation
IKZF5	4,24E-06	0,03	Elongation
DIEXF	4,57E-06	0,03	Elongation
URM1	4,62E-06	0,04	Elongation
FAM204A	4,66E-06	0,03	Elongation
YME1L1	4,72E-06	0,04	Elongation
WDR36	4,85E-06	0,04	Elongation
ARF3	5,25E-06	0,04	Elongation
PANK3	5,25E-06	0,05	Elongation
LRRC57	5,56E-06	0,04	Elongation
RAB18	6,57E-06	0,03	Elongation
MIB1	7,33E-06	-0,02	Shortening
AFF4	7,33E-06	0,05	Elongation
PRRC2B	7,33E-06	0,04	Elongation
TMX4	8,33E-06	0,03	Elongation
HSPA4	8,33E-06	0,04	Elongation
ICMT	8,88E-06	0,06	Elongation
PDCD6IP	8,88E-06	0,04	Elongation

EIF4E3	9,43E-06	0,06	Elongation
S100PBP	1,01E-05	0,04	Elongation
IMPAD1	1,06E-05	-0,02	Shortening
TAF1C	1,09E-05	0,04	Elongation
PIP4K2B	1,17E-05	0,06	Elongation
PDE11A	1,21E-05	0,04	Elongation
CADM2	1,21E-05	-0,04	Shortening
SRSF12	1,23E-05	0,02	Elongation
CISD1	1,38E-05	-0,02	Shortening
MRPL42	1,38E-05	0,03	Elongation
KPNA1	1,42E-05	0,03	Elongation
HS2ST1	1,48E-05	0,04	Elongation
SFXN1	1,72E-05	0,02	Elongation
SCN3A	1,74E-05	0,04	Elongation
DUSP7	1,74E-05	0,07	Elongation
NUCKS1	1,77E-05	-0,04	Shortening
MTMR9	2,22E-05	0,03	Elongation
MAGT1	2,29E-05	0,03	Elongation
NMT1	2,35E-05	0,04	Elongation
GXYLT1	2,39E-05	0,05	Elongation
QPRT	2,52E-05	0,03	Elongation
CCDC71L	2,52E-05	0,06	Elongation
TMEM261	2,60E-05	0,03	Elongation
FNTB	3,36E-05	0,04	Elongation
TSPAN9	3,64E-05	0,05	Elongation
EXOC5	3,68E-05	0,04	Elongation
HSBP1	3,73E-05	0,03	Elongation
RPS6KA3	3,81E-05	0,04	Elongation
ELAVL4	3,93E-05	-0,02	Shortening
PTBP3	3,98E-05	0,03	Elongation
TMEM50A	4,31E-05	0,07	Elongation
PRRG1	4,74E-05	-0,03	Shortening
CREB1	4,79E-05	0,05	Elongation
ARL3	4,94E-05	0,02	Elongation
SNU13	5,47E-05	0,03	Elongation
GRPEL2	6,01E-05	0,07	Elongation
VPS39	6,17E-05	0,03	Elongation
PGRMC2	6,21E-05	0,02	Elongation
ELAVL1	6,76E-05	-0,08	Shortening
DYM	6,92E-05	-0,02	Shortening
MID1	7,14E-05	-0,02	Shortening
DERL1	7,53E-05	0,04	Elongation
MAPK6	7,82E-05	0,04	Elongation
CLCN3	8,07E-05	0,02	Elongation
PTK2	8,79E-05	0,02	Elongation
VLDLR	8,80E-05	-0,03	Shortening
ABL2	9,81E-05	0,05	Elongation
NMNAT2	1,00E-04	0,04	Elongation
THSD4	1,06E-04	0,07	Elongation
ANKRD40	1,06E-04	0,04	Elongation

HNRNPD	1,10E-04	-0,02	Shortening
WDR12	1,15E-04	0,03	Elongation
DLC1	1,15E-04	0,02	Elongation
KIAA1549	1,39E-04	0,02	Elongation
ATG4B	1,39E-04	0,04	Elongation
DLAT	1,45E-04	0,04	Elongation
TGFBRAP1	1,55E-04	0,02	Elongation
PROSER1	1,56E-04	0,03	Elongation
TMEM237	1,61E-04	0,04	Elongation
PRELID3B	1,61E-04	0,03	Elongation
ZFYVE16	1,62E-04	0,03	Elongation
PEX13	1,65E-04	0,03	Elongation
RBM15B	1,77E-04	0,02	Elongation
ATXN3	1,80E-04	0,02	Elongation
EPB41L1	1,92E-04	0,03	Elongation
C8orf33	1,97E-04	0,02	Elongation
SPAG9	2,02E-04	0,03	Elongation
EPT1	2,02E-04	0,04	Elongation
GTPBP4	2,08E-04	0,03	Elongation
UBA2	2,10E-04	-0,02	Shortening
AMMECR1L	2,57E-04	0,04	Elongation
TIMM13	2,80E-04	0,02	Elongation
IRF2BP2	3,01E-04	0,03	Elongation
FAM124A	3,02E-04	0,03	Elongation
LRRC8B	3,08E-04	-0,02	Shortening
SRSF8	3,08E-04	0,02	Elongation
ZNF431	3,16E-04	-0,03	Shortening
ZMAT3	3,19E-04	-0,04	Shortening
UBE2K	3,28E-04	-0,02	Shortening
RHOU	3,30E-04	0,05	Elongation
TRMT61A	3,79E-04	-0,04	Shortening
ZNF548	3,80E-04	0,04	Elongation
GTF2F2	3,93E-04	0,02	Elongation
CHST15	4,32E-04	0,04	Elongation
TRIQK	4,52E-04	0,02	Elongation
GOLPH3L	4,83E-04	0,02	Elongation
METTL7A	5,08E-04	0,07	Elongation
VPS13D	5,12E-04	0,03	Elongation
SLF2	5,12E-04	0,03	Elongation
PYGO1	5,45E-04	0,04	Elongation
LIFR	5,58E-04	0,02	Elongation
TM9SF3	6,16E-04	0,03	Elongation
LINC01420	6,34E-04	-0,02	Shortening
MYEF2	6,34E-04	0,03	Elongation
CCT5	6,73E-04	-0,02	Shortening
SUPT4H1	7,47E-04	0,02	Elongation
UQCC1	7,89E-04	0,02	Elongation
SPPL2A	8,07E-04	0,02	Elongation
CORO2A	8,11E-04	0,04	Elongation
SENP5	8,72E-04	-0,03	Shortening

GDF11	8,81E-04	0,04	Elongation
ZNF598	9,30E-04	0,03	Elongation
BAG5	9,80E-04	-0,02	Shortening
PEX19	1,00E-03	0,02	Elongation
SLC12A2	1,11E-03	-0,04	Shortening
RBL1	1,13E-03	0,03	Elongation
DYRK1A	1,22E-03	0,03	Elongation
SLC30A5	1,32E-03	-0,02	Shortening
ENAH	1,41E-03	-0,01	Shortening
ING1	1,45E-03	0,03	Elongation
KATNAL1	1,47E-03	0,03	Elongation
OSBPL3	1,52E-03	0,04	Elongation
RERE	1,53E-03	0,03	Elongation
RHBDD1	1,62E-03	0,03	Elongation
ATP11A	1,68E-03	0,06	Elongation
SEPHS1	1,88E-03	0,02	Elongation
KIAA1468	1,89E-03	0,02	Elongation
IFT80	1,94E-03	0,02	Elongation
TOR1AIP2	1,94E-03	-0,01	Shortening
ANKRD9	1,95E-03	0,03	Elongation
EMC3	2,03E-03	0,02	Elongation
MDM2	2,15E-03	-0,02	Shortening
PURA	2,15E-03	-0,02	Shortening
YTHDF1	2,17E-03	0,03	Elongation
DCTN2	2,23E-03	-0,01	Shortening
MAP3K1	2,40E-03	-0,02	Shortening
ATPAF1	2,40E-03	0,02	Elongation
MLLT1	2,49E-03	-0,04	Shortening
WDR33	2,53E-03	-0,03	Shortening
RAB35	2,61E-03	0,01	Elongation
SARM1	2,79E-03	0,03	Elongation
FAM210A	2,90E-03	0,03	Elongation
NFATC2IP	2,90E-03	0,02	Elongation
TUBB	2,90E-03	-0,02	Shortening
HSPA13	3,04E-03	0,03	Elongation
GDE1	3,28E-03	0,02	Elongation
URB1	3,32E-03	-0,03	Shortening
ZNRF1	3,41E-03	0,03	Elongation
TRAK2	3,41E-03	0,02	Elongation
ALDH1B1	3,51E-03	-0,02	Shortening
JRK	3,58E-03	0,04	Elongation
TMEM170A	3,60E-03	-0,02	Shortening
ANKRD6	4,10E-03	0,03	Elongation
MAN1A2	4,13E-03	-0,03	Shortening
FAM208A	4,13E-03	0,03	Elongation
KCNK3	4,40E-03	0,02	Elongation
ABHD13	4,70E-03	-0,02	Shortening
CREBL2	4,77E-03	-0,02	Shortening
USP7	4,96E-03	0,02	Elongation
HN1	5,05E-03	0,01	Elongation

MYO5A	5,21E-03	0,02	Elongation
ECHDC1	5,47E-03	0,02	Elongation
CLOCK	5,57E-03	0,02	Elongation
ACOX1	5,64E-03	-0,01	Shortening
PDS5B	5,77E-03	0,02	Elongation
ZNF10	6,02E-03	0,03	Elongation
CAMK2N1	6,11E-03	-0,02	Shortening
UCHL5	6,26E-03	0,02	Elongation
HDAC4	6,26E-03	0,03	Elongation
RNF150	6,51E-03	-0,03	Shortening
PDXK	6,81E-03	0,04	Elongation
SCN8A	7,60E-03	0,02	Elongation
DOK4	8,30E-03	0,02	Elongation
FARP1	8,59E-03	0,02	Elongation
SMC5	8,59E-03	0,02	Elongation
OGT	8,67E-03	0,03	Elongation
PDLIM5	9,50E-03	0,02	Elongation
YPEL1	9,95E-03	-0,02	Shortening
IL17RD	1,05E-02	0,03	Elongation
CAB39L	1,07E-02	0,02	Elongation
FBXW7	1,07E-02	0,02	Elongation
RBPJ	1,09E-02	-0,01	Shortening
EFNB2	1,10E-02	0,02	Elongation
ATF6	1,12E-02	0,01	Elongation
NAB1	1,16E-02	0,03	Elongation
EPHB2	1,19E-02	0,03	Elongation
KIAA0930	1,19E-02	0,02	Elongation
AP3S2	1,20E-02	0,01	Elongation
SSBP2	1,28E-02	0,02	Elongation
GATAD1	1,30E-02	-0,01	Shortening
DDX18	1,32E-02	0,02	Elongation
ATMIN	1,39E-02	0,02	Elongation
CDC42EP3	1,40E-02	0,03	Elongation
CCDC125	1,43E-02	-0,02	Shortening
IER3IP1	1,48E-02	0,01	Elongation
BID	1,48E-02	-0,01	Shortening
OGFOD3	1,49E-02	-0,02	Shortening
SLU7	1,54E-02	0,02	Elongation
TLE4	1,54E-02	0,03	Elongation
ASAP1	1,68E-02	0,01	Elongation
ANP32A	1,71E-02	0,01	Elongation
TADA2B	1,75E-02	0,02	Elongation
LINC01128	1,77E-02	-0,02	Shortening
TLE3	1,79E-02	0,02	Elongation
RNF24	1,79E-02	0,01	Elongation
C3orf17	1,79E-02	0,02	Elongation
SLC9A8	1,81E-02	0,04	Elongation
TMTC3	1,87E-02	-0,02	Shortening
BECN1	1,89E-02	0,02	Elongation
PTCD3	1,96E-02	-0,04	Shortening

WIPF2	2,00E-02	0,02	Elongation
USP31	2,04E-02	-0,02	Shortening
CTDSPL2	2,14E-02	-0,03	Shortening
C11orf57	2,15E-02	-0,02	Shortening
PPP2R2A	2,15E-02	0,02	Elongation
ADGRL1	2,19E-02	-0,01	Shortening
HNRNPA3	2,39E-02	0,01	Elongation
RABIF	2,45E-02	0,01	Elongation
PAICS	2,45E-02	0,02	Elongation
UQCR11	2,48E-02	0,01	Elongation
NCS1	2,51E-02	0,02	Elongation
PRR3	2,52E-02	-0,02	Shortening
PDS5A	2,69E-02	0,01	Elongation
ASB6	2,72E-02	0,01	Elongation
KDSR	2,73E-02	0,01	Elongation
P2RX7	2,77E-02	-0,02	Shortening
MALT1	2,78E-02	0,01	Elongation
NAA50	2,89E-02	0,01	Elongation
METAP2	2,99E-02	0,01	Elongation
PI4K2A	3,02E-02	0,01	Elongation
HOOK3	3,05E-02	0,01	Elongation
ADAL	3,05E-02	-0,02	Shortening
GPBP1	3,10E-02	0,01	Elongation
CEP78	3,18E-02	0,02	Elongation
JADE2	3,24E-02	0,02	Elongation
FCF1	3,24E-02	-0,02	Shortening
PHF20	3,31E-02	0,02	Elongation
TRIM24	3,42E-02	0,01	Elongation
CALD1	3,42E-02	0,01	Elongation
SOCS4	3,47E-02	-0,02	Shortening
KCNQ2	3,53E-02	-0,04	Shortening
MAPK1IP1L	3,60E-02	-0,01	Shortening
DUSP1	3,67E-02	0,02	Elongation
VPS41	3,69E-02	0,02	Elongation
SLIT3	3,71E-02	-0,03	Shortening
NUDCD3	3,78E-02	-0,01	Shortening
CPD	3,80E-02	0,01	Elongation
MLX	3,80E-02	0,02	Elongation
AMD1	3,95E-02	-0,01	Shortening
NFIB	4,20E-02	-0,01	Shortening
MYLK	4,37E-02	-0,01	Shortening
IFIT5	4,58E-02	0,01	Elongation
MTMR6	4,58E-02	0,02	Elongation
TBC1D16	4,58E-02	-0,01	Shortening
MZT1	4,65E-02	0,02	Elongation
TECPR2	4,66E-02	-0,02	Shortening
CDH4	4,79E-02	-0,01	Shortening
SCG3	4,83E-02	-0,01	Shortening
WAPL	5,06E-02	0,02	NO TREND
STK17B	5,16E-02	0,02	NO TREND

AKAP13	5,17E-02	0,03	NO TREND
ZBTB2	5,33E-02	0,01	NO TREND
DSTYK	5,69E-02	0,01	NO TREND
QKI	5,69E-02	-0,02	NO TREND
RRM2	5,76E-02	0,02	NO TREND
SMC1A	5,85E-02	0,02	NO TREND
FASTKD2	5,88E-02	-0,01	NO TREND
RGP1	5,93E-02	0,02	NO TREND
ZNF276	5,93E-02	0,01	NO TREND
PARM1	6,21E-02	0,02	NO TREND
ORC4	6,25E-02	0,01	NO TREND
PNO1	6,30E-02	0,01	NO TREND
WDR26	6,69E-02	0,01	NO TREND
STK17A	6,73E-02	-0,02	NO TREND
FAM134A	6,81E-02	0,02	NO TREND
TMEM39A	6,82E-02	-0,01	NO TREND
ZFAND5	6,90E-02	0,02	NO TREND
TIA1	6,91E-02	-0,01	NO TREND
MRPL30	6,94E-02	0,02	NO TREND
ONECUT2	6,95E-02	0,02	NO TREND
ZMIZ2	7,06E-02	0,02	NO TREND
TMEM64	7,06E-02	-0,02	NO TREND
MPP2	7,14E-02	0,02	NO TREND
MT-RNR2	7,25E-02	-0,02	NO TREND
STC2	7,34E-02	0,01	NO TREND
SIKE1	7,37E-02	0,02	NO TREND
VEZF1	7,57E-02	0,01	NO TREND
CLASP2	7,80E-02	0,01	NO TREND
TJP1	7,87E-02	0,03	NO TREND
CDC42SE1	7,91E-02	0,01	NO TREND
MED28	7,93E-02	0,02	NO TREND
GTF3C4	8,31E-02	0,02	NO TREND
CSNK1A1	8,34E-02	0,01	NO TREND
CCDC127	8,43E-02	0,02	NO TREND
EMP2	8,47E-02	-0,01	NO TREND
MARCH5	8,68E-02	-0,01	NO TREND
MYCBP	8,74E-02	0,01	NO TREND
SREBF2	8,74E-02	0,01	NO TREND
GNPNAT1	8,81E-02	-0,01	NO TREND
USP14	8,85E-02	0,01	NO TREND
GCLM	8,86E-02	0,01	NO TREND
SETDB2	8,93E-02	-0,01	NO TREND
ARHGAP26	8,93E-02	0,02	NO TREND
OTUD7B	9,08E-02	-0,02	NO TREND
TRIB2	9,54E-02	0,01	NO TREND
AMFR	9,79E-02	-0,02	NO TREND
NCOA5	9,92E-02	0,01	NO TREND
TTC28	9,92E-02	-0,01	NO TREND
ERO1L	1,01E-01	0,01	NO TREND
B3GALNT1	1,02E-01	0,02	NO TREND

PPP6C	1,02E-01	0,01	NO TREND
CBX5	1,06E-01	0,02	NO TREND
PHAX	1,06E-01	-0,01	NO TREND
VANGL1	1,10E-01	-0,01	NO TREND
ADNP	1,13E-01	-0,01	NO TREND
RIT1	1,14E-01	-0,01	NO TREND
MRPL50	1,15E-01	0,01	NO TREND
SEC62	1,16E-01	-0,01	NO TREND
ZBTB26	1,16E-01	0,02	NO TREND
H3F3B	1,17E-01	0,01	NO TREND
CHD3	1,18E-01	0,01	NO TREND
MRPL57	1,19E-01	0,01	NO TREND
KCTD15	1,20E-01	-0,01	NO TREND
HS3ST5	1,22E-01	-0,01	NO TREND
RAB3D	1,22E-01	0,01	NO TREND
SIPA1L1	1,26E-01	-0,01	NO TREND
LRPAP1	1,26E-01	0,02	NO TREND
SF3A1	1,26E-01	-0,01	NO TREND
NHLRC2	1,26E-01	-0,01	NO TREND
CTB-89H12.4	1,26E-01	0,01	NO TREND
EIF2S1	1,27E-01	0,01	NO TREND
MBD3	1,29E-01	-0,02	NO TREND
WDR77	1,29E-01	0,01	NO TREND
ERLIN2	1,29E-01	0,02	NO TREND
DLG1	1,32E-01	0,02	NO TREND
LSM14A	1,32E-01	0,01	NO TREND
LPGAT1	1,34E-01	-0,01	NO TREND
SSH1	1,34E-01	0,01	NO TREND
TMEM97	1,34E-01	0,02	NO TREND
TP53RK	1,34E-01	0,01	NO TREND
RRP1B	1,34E-01	-0,02	NO TREND
UBL3	1,34E-01	0,01	NO TREND
ADI1	1,34E-01	0,02	NO TREND
FOXP1	1,34E-01	0,01	NO TREND
JAKMIP2	1,34E-01	0,01	NO TREND
WSB2	1,37E-01	0,01	NO TREND
SMAD9	1,38E-01	0,01	NO TREND
ACAP2	1,40E-01	0,01	NO TREND
MRPS10	1,41E-01	0,02	NO TREND
MMP16	1,43E-01	0,02	NO TREND
C2CD2L	1,43E-01	-0,01	NO TREND
GCLC	1,47E-01	-0,01	NO TREND
TIPRL	1,47E-01	-0,01	NO TREND
AKIP1	1,48E-01	0,01	NO TREND
RRP15	1,49E-01	0,01	NO TREND
FBXW8	1,51E-01	0,01	NO TREND
YOD1	1,54E-01	0,01	NO TREND
UBTD2	1,56E-01	0,01	NO TREND
PAQR8	1,56E-01	-0,01	NO TREND
TNS1	1,61E-01	0,01	NO TREND

SMKR1	1,62E-01	-0,01	NO TREND
UBE2H	1,62E-01	0,01	NO TREND
SFT2D3	1,70E-01	0,01	NO TREND
DCAF10	1,75E-01	0,01	NO TREND
BRWD1	1,78E-01	-0,01	NO TREND
RAB4A	1,85E-01	0,01	NO TREND
SOAT1	1,87E-01	0,02	NO TREND
CBX6	1,87E-01	-0,01	NO TREND
PLBD2	1,90E-01	-0,01	NO TREND
METTL8	1,96E-01	0,00	NO TREND
XPR1	1,96E-01	0,01	NO TREND
GGCX	2,04E-01	-0,01	NO TREND
CHTOP	2,04E-01	0,01	NO TREND
GFER	2,05E-01	-0,01	NO TREND
TBX3	2,15E-01	0,02	NO TREND
PGBD4	2,15E-01	0,01	NO TREND
INSIG1	2,15E-01	0,01	NO TREND
EPM2AIP1	2,15E-01	-0,01	NO TREND
ERCC6L2	2,18E-01	-0,01	NO TREND
RFFL	2,20E-01	0,01	NO TREND
RABL3	2,24E-01	0,01	NO TREND
CORO1C	2,24E-01	0,01	NO TREND
HNRNPA0	2,27E-01	0,01	NO TREND
PLA2G12A	2,35E-01	-0,01	NO TREND
PHF21A	2,36E-01	-0,01	NO TREND
TFAM	2,39E-01	0,01	NO TREND
SLC38A1	2,39E-01	-0,01	NO TREND
NGRN	2,39E-01	-0,01	NO TREND
SIN3A	2,40E-01	-0,01	NO TREND
JKAMP	2,42E-01	0,01	NO TREND
GRIK2	2,43E-01	0,01	NO TREND
NDUFC2	2,44E-01	-0,01	NO TREND
ROBO2	2,44E-01	-0,01	NO TREND
AGO2	2,45E-01	0,01	NO TREND
UBE2Z	2,49E-01	-0,01	NO TREND
GPR27	2,55E-01	0,02	NO TREND
FZD3	2,56E-01	-0,01	NO TREND
ME2	2,62E-01	0,01	NO TREND
ADAM12	2,64E-01	-0,02	NO TREND
ARPIN	2,68E-01	0,01	NO TREND
GLS	2,69E-01	0,01	NO TREND
AP1S2	2,72E-01	-0,01	NO TREND
WNK1	2,73E-01	0,01	NO TREND
SCRT1	2,73E-01	0,01	NO TREND
EP400	2,79E-01	0,01	NO TREND
AGAP1	2,84E-01	-0,01	NO TREND
PPP2R2D	2,89E-01	0,01	NO TREND
TOR1AIP1	2,93E-01	0,01	NO TREND
FKBP14	2,94E-01	-0,01	NO TREND
CNOT6	2,97E-01	0,01	NO TREND

BLOC1S2	2,99E-01	0,01	NO TREND
SLC16A7	3,00E-01	0,01	NO TREND
SSTR2	3,01E-01	-0,01	NO TREND
CDCA4	3,01E-01	0,01	NO TREND
CNIH1	3,03E-01	0,01	NO TREND
ZBTB21	3,13E-01	-0,01	NO TREND
CNTNAP2	3,13E-01	-0,01	NO TREND
PPP2R5E	3,14E-01	0,01	NO TREND
NOL9	3,15E-01	0,01	NO TREND
ZKSCAN5	3,15E-01	-0,01	NO TREND
NUDCD1	3,36E-01	-0,01	NO TREND
TTC30A	3,38E-01	0,01	NO TREND
ATP6V1C1	3,39E-01	0,01	NO TREND
ARNT	3,50E-01	-0,01	NO TREND
PDZD8	3,57E-01	0,01	NO TREND
TIMM21	3,62E-01	0,00	NO TREND
COMMD6	3,63E-01	0,00	NO TREND
ANKIB1	3,70E-01	0,00	NO TREND
STOX2	3,78E-01	0,01	NO TREND
TBC1D9B	3,83E-01	0,00	NO TREND
DCBLD2	3,91E-01	-0,01	NO TREND
SLC38A2	3,94E-01	0,01	NO TREND
PRKAB2	3,97E-01	0,01	NO TREND
SLC30A6	3,97E-01	-0,01	NO TREND
PMEPA1	3,97E-01	0,00	NO TREND
BRI3BP	4,04E-01	-0,01	NO TREND
THSD7A	4,14E-01	-0,01	NO TREND
TRIM25	4,19E-01	0,01	NO TREND
CCDC88A	4,32E-01	-0,01	NO TREND
RSBN1L	4,32E-01	-0,01	NO TREND
ZNF134	4,37E-01	-0,01	NO TREND
SNX3	4,38E-01	0,00	NO TREND
FRA10AC1	4,39E-01	0,00	NO TREND
FAM104A	4,42E-01	0,01	NO TREND
GOLGB1	4,47E-01	0,00	NO TREND
ZNF48	4,47E-01	0,01	NO TREND
RAD23B	4,47E-01	0,00	NO TREND
SRSF1	4,49E-01	0,01	NO TREND
SLC35B4	4,50E-01	0,00	NO TREND
FOXRED2	4,52E-01	0,00	NO TREND
HIPK1	4,54E-01	-0,01	NO TREND
PGM2L1	4,65E-01	0,01	NO TREND
RC3H1	4,77E-01	-0,01	NO TREND
PFAS	4,80E-01	0,01	NO TREND
USP25	4,80E-01	0,00	NO TREND
SLC8A2	4,88E-01	-0,01	NO TREND
FOSL2	4,91E-01	-0,01	NO TREND
ERO1A	5,05E-01	-0,01	NO TREND
TIFA	5,09E-01	0,01	NO TREND
PKIA	5,09E-01	-0,01	NO TREND

AHNAK2	5,13E-01	0,01	NO TREND
IDI1	5,15E-01	0,00	NO TREND
SPATA2	5,15E-01	0,01	NO TREND
CFL2	5,17E-01	0,01	NO TREND
EIF2A	5,17E-01	0,01	NO TREND
HMGCS1	5,41E-01	0,00	NO TREND
PI4KB	5,43E-01	0,00	NO TREND
XRCC2	5,45E-01	-0,01	NO TREND
QSOX1	5,61E-01	0,00	NO TREND
CAMK4	5,61E-01	-0,01	NO TREND
RALGAPA2	5,63E-01	0,00	NO TREND
NDUFAF4	5,65E-01	0,01	NO TREND
PHLDA1	5,65E-01	0,00	NO TREND
MCFD2	5,69E-01	0,00	NO TREND
DYNC1LI2	5,71E-01	0,00	NO TREND
RSL1D1	5,72E-01	0,00	NO TREND
RNF41	5,77E-01	0,00	NO TREND
GABARAPL2	5,82E-01	0,00	NO TREND
ARL6IP1	5,92E-01	0,00	NO TREND
GLG1	5,92E-01	0,00	NO TREND
B3GNT9	6,09E-01	-0,01	NO TREND
SLC35F6	6,15E-01	0,00	NO TREND
MLF1	6,24E-01	0,00	NO TREND
NUP43	6,24E-01	-0,01	NO TREND
OSBPL6	6,26E-01	0,00	NO TREND
FBRSL1	6,30E-01	-0,01	NO TREND
PEA15	6,30E-01	0,00	NO TREND
DAG1	6,42E-01	0,00	NO TREND
RAD1	6,42E-01	0,00	NO TREND
RAB14	6,55E-01	0,00	NO TREND
GAPVD1	6,72E-01	0,00	NO TREND
CCNL2	6,79E-01	0,00	NO TREND
HSD17B11	6,98E-01	0,00	NO TREND
CPPED1	7,06E-01	0,00	NO TREND
PTGS1	7,06E-01	0,00	NO TREND
SLC25A33	7,25E-01	0,00	NO TREND
DNAJC10	7,25E-01	0,00	NO TREND
TRIP12	7,29E-01	0,00	NO TREND
AP3B1	7,43E-01	0,00	NO TREND
TACC1	7,47E-01	0,00	NO TREND
CALM1	7,57E-01	0,00	NO TREND
LYRM4	7,61E-01	0,00	NO TREND
CCND1	7,63E-01	0,00	NO TREND
MTMR2	7,63E-01	0,00	NO TREND
NSD1	7,63E-01	0,00	NO TREND
MED17	7,67E-01	0,00	NO TREND
ATG13	7,72E-01	0,00	NO TREND
PPM1E	7,79E-01	0,00	NO TREND
ZNF24	7,81E-01	0,00	NO TREND
MDM4	7,85E-01	0,00	NO TREND

CNKSR3	7,85E-01	0,00	NO TREND
ACOX3	7,87E-01	0,00	NO TREND
TPM3	7,92E-01	0,00	NO TREND
CCDC25	7,95E-01	0,00	NO TREND
BCL7A	8,20E-01	0,00	NO TREND
TMED2	8,33E-01	0,00	NO TREND
CX3CL1	8,36E-01	0,00	NO TREND
POGZ	8,41E-01	0,00	NO TREND
LSAMP	8,42E-01	0,00	NO TREND
GNAQ	8,43E-01	0,00	NO TREND
C14orf132	8,48E-01	0,00	NO TREND
EDC3	8,51E-01	0,00	NO TREND
TFCP2	8,61E-01	0,00	NO TREND
CCDC47	8,64E-01	0,00	NO TREND
ERGIC1	8,72E-01	0,00	NO TREND
C19orf12	8,73E-01	0,00	NO TREND
KHNYN	8,84E-01	0,00	NO TREND
CSDE1	8,84E-01	0,00	NO TREND
MPLKIP	8,84E-01	0,00	NO TREND
EXTL3	8,90E-01	0,00	NO TREND
FKBP5	9,00E-01	0,00	NO TREND
METTL14	9,06E-01	0,00	NO TREND
CHRM3	9,07E-01	0,00	NO TREND
RPP14	9,09E-01	0,00	NO TREND
MARCH3	9,09E-01	0,00	NO TREND
ADAM17	9,25E-01	0,00	NO TREND
CUL4A	9,32E-01	0,00	NO TREND
AGPAT3	9,35E-01	0,00	NO TREND
SETX	9,35E-01	0,00	NO TREND
PSMB2	9,39E-01	0,00	NO TREND
ALDH2	9,44E-01	0,00	NO TREND
TRAF1	9,51E-01	0,00	NO TREND
TMEM18	9,77E-01	0,00	NO TREND
NDUFV3	9,91E-01	0,00	NO TREND
CSTF2T	9,97E-01	0,00	NO TREND

Supplementary Table 6

Area Under Curve (AUC) values for TREND alterations of detectable genes belonging to the neurodifferentiation TREND-operon (Supplementary Table 4, Fig. 3b)

AUCs were calculated using pROC R package (PMID: 21414208).

Respective Receiver Operating Characteristic (ROC) curves for chosen genes are shown in Fig 7b.

Gene Name	All tumor samples Death AUC n=493	non MYCN amplified tumor samples Death AUC n=401	All tumor samples High-Risk AUC n=493	non MYCN amplified tumor samples High-Risk AUC n=401
PLEKHA6	0,81	0,79	0,85	0,79
PVRL1	0,81	0,79	0,87	0,83
GNG2	0,73	0,72	0,81	0,75
ELP2	0,73	0,69	0,71	0,62
AES	0,73	0,64	0,72	0,63
AKT2	0,72	0,67	0,71	0,63
RBM17	0,7	0,7	0,7	0,67
IGF1R	0,69	0,67	0,73	0,68
GNB1	0,68	0,7	0,74	0,71
NMT1	0,68	0,72	0,66	0,69
TM9SF3	0,64	0,75	0,69	0,74
PDXK	0,62	0,59	0,68	0,65
CD2BP2	0,57	0,41	0,58	0,36
ASB6	0,56	0,52	0,54	0,48
HNRNPA3	0,5	0,5	0,52	0,53
METAP2	0,49	0,51	0,51	0,47
UNC5C	0,47	0,49	0,53	0,64

Supplementary Table 7

P-values of bootstrap comparison between the predictive power of established risk marker expression and combined TREND-patterns

Stratifier	Risk marker	All samples	Non MYCN amp samples
Death	MYCN	$1.6 * 10^{-4}$	$3.1 * 10^{-8}$
Death	ALK	$7.9 * 10^{-5}$	$1.2 * 10^{-3}$
High Risk	MYCN	$2.6 * 10^{-7}$	$4.0 * 10^{-14}$
High Risk	ALK	$2.3 * 10^{-9}$	$2.3 * 10^{-5}$
